# Fibrin drives thromboinflammation and neuropathology in COVID-19

**DOI:** 10.1038/s41586-024-07873-4

**Published:** 2024-08-28

**Authors:** Jae Kyu Ryu, Zhaoqi Yan, Mauricio Montano, Elif G. Sozmen, Karuna Dixit, Rahul K. Suryawanshi, Yusuke Matsui, Ekram Helmy, Prashant Kaushal, Sara K. Makanani, Thomas J. Deerinck, Anke Meyer-Franke, Pamela E. Rios Coronado, Troy N. Trevino, Min-Gyoung Shin, Reshmi Tognatta, Yixin Liu, Renaud Schuck, Lucas Le, Hisao Miyajima, Andrew S. Mendiola, Nikhita Arun, Brandon Guo, Taha Y. Taha, Ayushi Agrawal, Eilidh MacDonald, Oliver Aries, Aaron Yan, Olivia Weaver, Mark A. Petersen, Rosa Meza Acevedo, Maria del Pilar S. Alzamora, Reuben Thomas, Michela Traglia, Valentina L. Kouznetsova, Igor F. Tsigelny, Alexander R. Pico, Kristy Red-Horse, Mark H. Ellisman, Nevan J. Krogan, Mehdi Bouhaddou, Melanie Ott, Warner C. Greene, Katerina Akassoglou

**Affiliations:** 1 Center for Neurovascular Brain Immunology at Gladstone and UCSF, San Francisco, CA USA; 2 Gladstone Institute of Neurological Disease, San Francisco, CA USA; 3 Department of Neurology, Weill Institute for Neurosciences, University of California San Francisco, San Francisco, CA USA; 4 Gladstone Institute of Virology, San Francisco, CA USA; 5 Michael Hulton Center for HIV Cure Research at Gladstone, San Francisco, CA USA; 6 Department of Microbiology, Immunology and Molecular Genetics (MIMG), University of California Los Angeles, Los Angeles, CA USA; 7 Institute for Quantitative and Computational Biosciences (QCBio), University of California Los Angeles, Los Angeles, CA USA; 8 National Center for Microscopy and Imaging Research, Center for Research on Biological Systems, University of California San Diego, La Jolla, CA USA; 9 Department of Biology, Stanford University, Stanford, CA USA; 10 Gladstone Institute of Data Science and Biotechnology, San Francisco, CA USA; 11 Department of Pediatrics, University of California San Francisco, San Francisco, CA USA; 12 San Diego Supercomputer Center, University of California San Diego, La Jolla, CA USA; 13 CureScience Institute, San Diego, CA USA; 14 Department of Neurosciences, University of California San Diego, La Jolla, CA USA; 15 Institute for Stem Cell Biology and Regenerative Medicine, Stanford University School of Medicine, Stanford, CA USA; 16 Howard Hughes Medical Institute, Stanford University, Stanford, CA USA; 17 Department of Cellular and Molecular Pharmacology, University of California San Francisco, San Francisco, CA USA; 18 Quantitative Biosciences Institute (QBI), University of California San Francisco, San Francisco, CA USA; 19 COVID-19 Research Group (QCRG), University of California San Francisco, San Francisco, CA USA; 20 Department of Medicine, University of California, San Francisco, San Francisco, CA USA; 21 Chan Zuckerberg Biohub, San Francisco, CA USA; 22 Department of Microbiology and Immunology, University of California, San Francisco, San Francisco, CA USA

**Keywords:** Neuroimmunology, Biologics, Infection

## Abstract

Life-threatening thrombotic events and neurological symptoms are prevalent in COVID-19 and are persistent in patients with long COVID experiencing post-acute sequelae of SARS-CoV-2 infection^1–4^. Despite the clinical evidence^1,5–7^, the underlying mechanisms of coagulopathy in COVID-19 and its consequences in inflammation and neuropathology remain poorly understood and treatment options are insufficient. Fibrinogen, the central structural component of blood clots, is abundantly deposited in the lungs and brains of patients with COVID-19, correlates with disease severity and is a predictive biomarker for post-COVID-19 cognitive deficits^1,5,8–10^. Here we show that fibrin binds to the SARS-CoV-2 spike protein, forming proinflammatory blood clots that drive systemic thromboinflammation and neuropathology in COVID-19. Fibrin, acting through its inflammatory domain, is required for oxidative stress and macrophage activation in the lungs, whereas it suppresses natural killer cells, after SARS-CoV-2 infection. Fibrin promotes neuroinflammation and neuronal loss after infection, as well as innate immune activation in the brain and lungs independently of active infection. A monoclonal antibody targeting the inflammatory fibrin domain provides protection from microglial activation and neuronal injury, as well as from thromboinflammation in the lung after infection. Thus, fibrin drives inflammation and neuropathology in SARS-CoV-2 infection, and fibrin-targeting immunotherapy may represent a therapeutic intervention for patients with acute COVID-19 and long COVID.

## Abstract (web summary)

Fibrin drives inflammation and neuropathology in SARS-CoV-2 infection, and fibrin-targeting immunotherapy may represent a therapeutic intervention for patients with long COVID.

## Main

Long COVID has emerged as a central public health issue that remains an unmet clinical need^4^. Coagulation and neurological complications in COVID-19 can occur during acute infection and persist in long COVID causing morbidity and mortality^1–4,11^. Notably, coagulopathy also occurs in young patients with COVID-19 with mild infections, breakthrough infections and long COVID, and is associated with neurological complications^3–7,12^. Blood clots in patients with COVID-19 remain resistant to degradation despite adequate anticoagulation^1,13,14^. The prevalence and severity of coagulopathy and its correlations with the immune response and neurological complications in long COVID suggest as yet unknown mechanisms of COVID-19 pathogenesis.

Hypercoagulability in COVID-19 is associated with extensive fibrin deposition in inflamed lung and brain^8–10^. Fibrin is derived from the soluble blood protein fibrinogen after activation of coagulation and forms the central structural component of blood clots^15,16^. Fibrin is deposited at sites of vascular damage or blood–brain barrier (BBB) disruption, and is a key proinflammatory and prooxidant activator of the innate immune response in autoimmune, inflammatory and neurodegenerative diseases^15,17–21^. Neurovascular injury and reactive microglia are detected at sites of parenchymal fibrin deposition in brains of patients with COVID-19^8,9^. BBB disruption correlates with brain fog in long COVID, and increased plasma fibrinogen is a predictive biomarker of cognitive deficits after COVID-19^1,5,22^. However, the role of blood clots in COVID-19 inflammation and neurological changes remains largely unclear, and therapies to combat their effects are not readily available.

Here we provide evidence for a fundamental role of fibrinogen in the COVID-19 immune response and neuropathology, and identify a potential antibody-based strategy to combat the deleterious effects of abnormal blood clots in acute and long COVID.

## Fibrinogen binds to SARS-CoV-2 spike

**Fig. 1:**
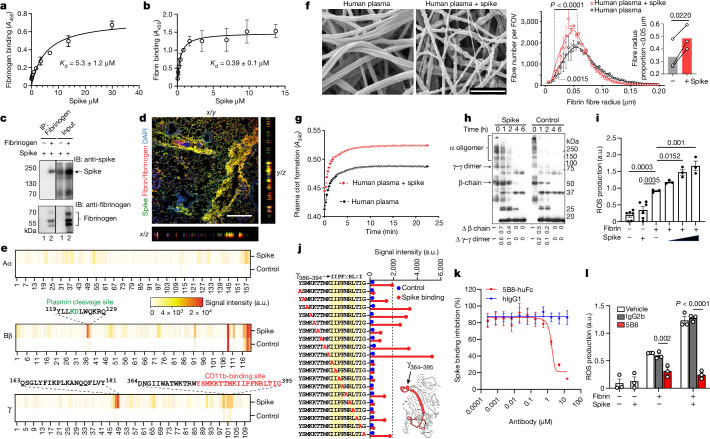
Fibrinogen interaction with SARS-CoV-2 spike. **a**,**b**, Binding enzyme-linked immunosorbent assay (ELISA) of spike to fibrinogen (**a**) or fibrin (**b**). *K*_d_, dissociation constant. *A*_450_, absorbance at 450 nm. **c**, Fibrinogen immunoprecipitation (IP) with spike. **d**, Spike and fibrinogen immunoreactivity in the lungs at 3 d.p.i. Representative of five Beta-infected WT mice. Scale bar, 300 μm. **e**, Peptide array of fibrinogen chains Aα, Bβ and γ blotted with spike. The binding signal intensity is shown (white to orange). **f**, Scanning electron microscopy (SEM) images and quantification of the fibrin clot fibre radius in human plasma with spike. The fibre radius distribution was determined in *n* = 25 (plasma) and *n* = 28 (plasma with spike) images from three biologically independent experiments (generalized linear mixed-effects model with Holmes multiple correction; Methods) and the fibre radius proportion (<0.05 µm) was determined from *n* = 3 biologically independent experiments (two-sided paired *t*-test; Methods). Scale bar, 1 µm. FOV, field of view. **g**, The turbidity of fibrin polymerization with spike in human plasma. **h**, Immunoblot (IB) analysis of fibrin degradation by plasmin representative from five (0, 2 and 4 h) or three (1 and 6 h) biologically independent experiments. **i**, ROS in BMDMs stimulated with fibrin and/or spike. *n* = 6 (unstimulated and spike) and *n* = 3 (fibrin or fibrin with spike) biologically independent experiments. a.u., arbitrary units. **j**, Fibrin γC domain and spike-binding epitope γ_364–395_ (red). Alanine scanning of γ_377–395_ blotted with His–spike. The binding of spike to Ala-substituted peptides is shown. The residues that are required for binding are indicated in yellow. **k**, Competitive ELISA of 5B8-huFc (5B8 with human IgG1 Fc region) or huIgG1 versus spike for binding to fibrin. *n* = 3 biologically independent experiments. **l**, ROS in BMDMs stimulated with fibrin and/or spike treated with 5B8 or IgG2b. *n* = 3 biologically independent experiments. Representative data of *n* = 3 (**a**–**c**) or *n* = 4 (**g**) biologically independent experiments. For **i** and **l**, statistical analysis was performed using one-way analysis of variance (ANOVA) with Tukey’s multiple-comparison test. Data are mean ± s.e.m. Gel source data are provided in Supplementary Fig. 1. Source Data

**Extended Data Fig. 1:**
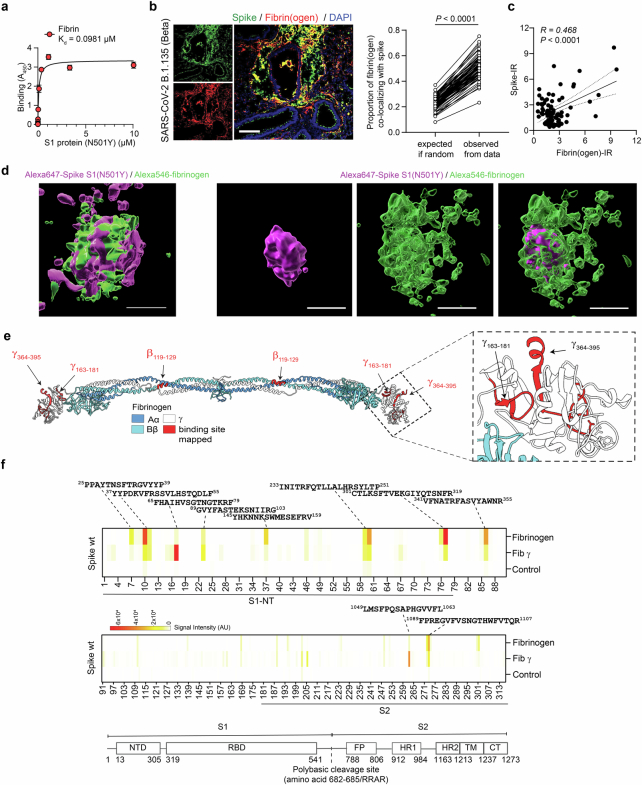
Fibrin interaction and colocalization with Spike. **a**, Binding ELISA of Spike S1(N501Y) to fibrin. Dissociation constants (K_d_). Representative curvefits from two independent biological experiments in duplicates. **b**, Spike overlap with perivascular fibrin(ogen) deposition in lung of Beta-infected WT mice at 3 d.p.i. The 51% of the calculated proportion of fibrin that colocalizes with Spike protein is significantly higher than the 23% predicted if the correlation were random. Fisher’s exact test (two-tailed); *n* = 78 images from 5 mice (Methods). Representative confocal images are shown. Scale bar, 200 μm. **c**, Scatter plot of positive correlation of fibrinogen and Spike immunoreactivity in *n* = 78 images from 5 mice, Pearson correlation two-tailed (Methods). **d**. 3D reconstruction of light sheet acquisitions of whole lung tissue from an Alexa546-fibrinogen and Alexa647-Spike S1(N501Y)-injected WT mouse following 3DISCO tissue clearing. Two representative focal fibrinogen deposits from *n* = 3 mice were selected for 3D visualization. Volumetric rendering reveals close interactions between fibrinogen deposits (green) and trimeric spike (magenta), confirming colocalization. Scale bars, 100 μm (top), 300 μm (bottom). **e**, Fibrinogen crystal structure (PDB: 3GHG) with mapped peptides (red). Proximity of peptides γ_163-181_ and γ_364-395_ (inset). **f**, Peptide array mapping with immobilized peptides of SARS-CoV-2 Spike blotted with fibrinogen and fibrinogen γ chain. Heatmap of signal intensity showing binding sites (white-orange) within the S1-NT Spike domain. Key indicates fluorescence intensities signal values from low (white) to high (orange). Schematic indicating Spike domains and amino acid sequence. Source Data

Given that patients with COVID-19 have a higher frequency and severity of abnormal blood clots than other common respiratory viral infections^1,23^, we hypothesized that SARS-CoV-2 directly binds to fibrinogen, promoting blood clot formation and altering clot structure and function. A solid-phase binding assay revealed binding of fibrinogen and fibrin to the SARS-CoV-2 recombinant trimeric spike protein (spike) and to the spike S1(N501Y) mutant, which enhances SARS-CoV-2 transmission and binding to mouse angiotensin-converting enzyme 2 (ACE2)^24^ (Fig. 1a,b and Extended Data Fig. 1a). The affinity of spike binding to fibrin (390 nM for trimeric Wuhan spike and 98 nM for spike S1(N501Y)) was lower than that of spike binding to ACE2 (1–15 nM range)^24^. Fibrinogen immunoprecipitated with full-length recombinant trimeric spike (Fig. 1c). Fibrinogen and spike co-localized in the lungs after either intranasal (i.n.) infection of mice with mouse-proficient SARS-CoV-2 Beta (B.1.351) (Fig. 1d and Extended Data Fig. 1b,c) or intravenous (i.v.) co-injection, into wild-type (WT) mice, of Alexa 647–spike S1(N501Y) and Alexa 546–fibrinogen, as shown by 3D imaging of solvent-cleared organs (3DISCO)^20^ of cleared lungs (Extended Data Fig. 1d), suggesting that fibrin/fibrinogen and spike interact in solution and in tissues.

**Extended Data Fig. 2:**
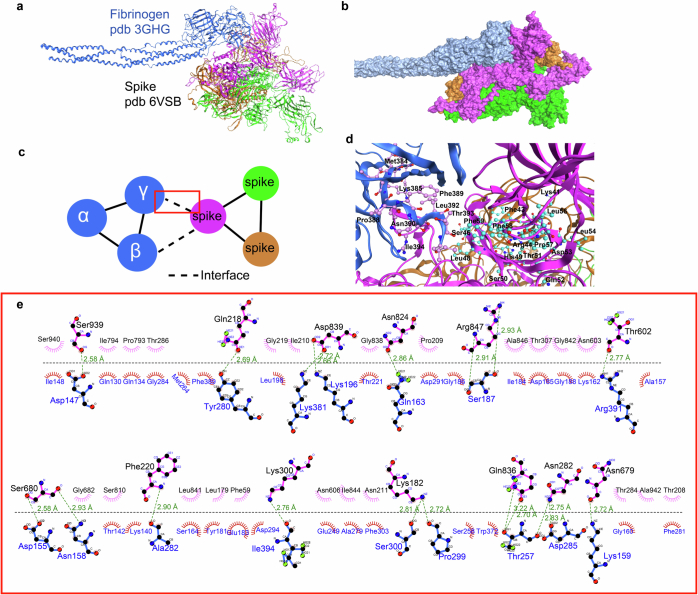
Computational modelling of fibrinogen-Spike protein interactions. **a**, Predicted computational complex model using crystal structure of fibrinogen (PDB ID: 3GHG) and cryoEM structure of Spike (PDB ID: 6VSB). **b**, Surface of this complex. **c**, Schematic representation of intermolecular interface of this predicted model. **d**, 3D representation of interface shown by side chain of the few residues involved in interaction. **e**, Intermolecular interface showing atom-wise interaction with all the residues involved in the interaction including a hydrogen bond highlighted in green dashed line using LigPlot +, v.2.2. https://www.ebi.ac.uk/thornton-srv/software/LigPlus/.

To identify spike-binding regions in fibrinogen, we generated a custom fibrinogen peptide array of 390 15-mer peptides overlapping by 11 amino acids, spanning the Aα, Bβ and γ chains (Fig. 1e and Supplementary Table 1). Hybridization with His-tagged trimeric spike identified three major binding sites in the Bβ and γ fibrinogen chains, namely Bβ_119–129_, which contains cleavage sites for the fibrinolytic serine protease plasmin^25^; γ_163–181_, of unknown function; and γ_364–395_, which encompasses the γ_377–395_ cryptic fibrinogen-binding site for complement receptor 3 that activates innate immune responses^15,26^ (Fig. 1e). Mapping the spike-binding peptides onto the fibrinogen crystal structure revealed proximity of the γ_163–181_ and γ_377–395_ peptides, suggesting that a 3D conformational epitope in the carboxy-terminal γ-chain of fibrinogen (γC domain) is involved in fibrinogen binding to spike (Extended Data Fig. 1e). Reverse mapping of fibrinogen binding on SARS-CoV-2 spike variants revealed binding sites spike_37–103_, spike_229–251_ within the N terminal domain (NTD) S1 subunit, spike_305–319_, spike_341–355_ within the receptor-binding domain (RBD) and spike_1049–1063_ within the S2 subunit (Extended Data Fig. 1f and Supplementary Table 2). Computational docking identified a model with the best docking energies with close association between fibrinogen γ_364–395_ and spike_37–103_ (Extended Data Fig. 2 and Supplementary Table 3).

**Extended Data Fig. 3:**
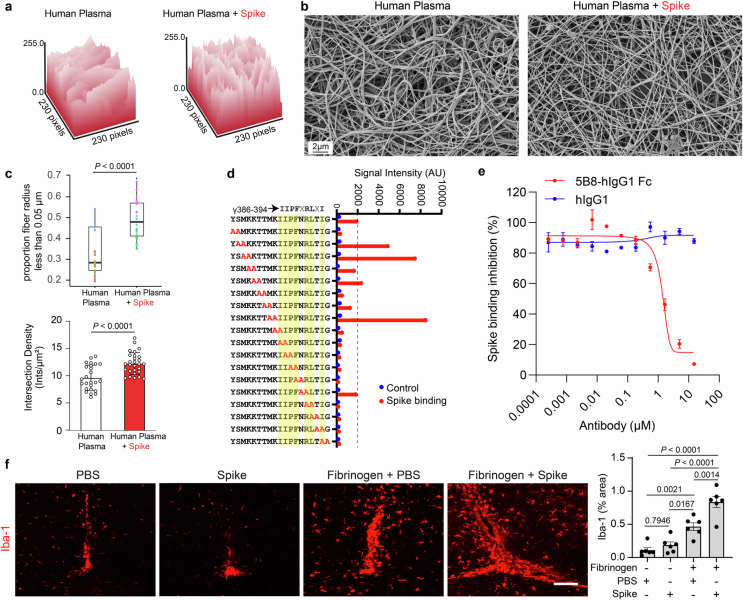
Fibrin clot ultrastructure and Spike protein interactions. **a**, Topographic visualization of fibrin fibre surface in SEM images of fibrin clots in healthy human donor plasma in the presence of Spike. **b**, SEM of fibrin clots in human plasma in the presence of Spike. x4000 magnification. Images representative of *n* = 3 independent biological replicates quantified in (**c**) and Fig. 1f. **c**, Fibre radius proportion less than 0.05 µm (boxplot) and intersection density (bar plot) in plasma or plasma with Spike. Generalized linear mixed effects model (boxplot) and two-sample two-sided Welch t-test (bar plot). *n* = 25 (plasma), *n* = 28 (plasma with Spike) images from *n* = 3 biologically independent experiments quantified in Fig. 1f. Images from biologically independent experiments are indicated by different colour dots in boxplot. Box indicates the interquartile range (IQR) and whiskers denote the 1.5 × IQR. **d**, Alanine scan mutagenesis peptide array. Fibrin peptide γ_377-395_ was subjected to double-alanine scanning mutagenesis and incubated with His-tagged recombinant Spike. Signal intensity bar graph of the binding of Spike to sequential Ala-Ala substituted peptides (red). Control signal is shown in blue. Residues with low signal intensity upon Ala-Ala substitution are required for binding and highlighted in yellow. **e**. ELISA of 5B8-huFc or huIgG1 isotype control pre-incubated with fibrin versus the Spike for binding to fibrin. Data are mean ± s.e.m from three biologically independent experiments. **f**. Iba-1 immunoreactivity in brain following stereotaxic co-injection of fibrinogen with PBS or Spike in WT mice. Scale bar, 50 µm. Data are from *n* = 6 mice per group. One-way ANOVA with Tukey’s multiple comparisons test. All data are mean ± s.e.m. Source Data

We next tested whether spike interferes with the polymerization, degradation and inflammatory properties of fibrin. Incubation of spike with healthy donor plasma in the presence of thrombin, which is elevated during COVID-19^1^, resulted in altered clot structure shown by scanning electron microscopy (SEM) and increased turbidity of fibrin clot formation (Fig. 1f,g and Extended Data Fig. 3a–c). Incubation of spike with fibrin delayed plasmin degradation of both the β-chain and the γ–γ dimer (Fig. 1h), suggesting that spike delays fibrinolysis. These findings are consistent with the formation of dense fibrin clots with thin fibres in thromboembolic diseases and fibrinolysis-resistant blood clots in patients with COVID-19^1,13,23^. Notably, spike increased fibrin-induced release of reactive oxygen species (ROS) in a concentration-dependent manner in bone-marrow-derived macrophages (BMDMs), while spike alone did not have an effect (Fig. 1i), suggesting that the SARS-CoV-2 virus enhances fibrin-induced inflammation. Using alanine scanning mutagenesis, we found that spike interacts with amino acids 386–394 in the C terminus of the γ_377–395_ peptide (Fig. 1j and Extended Data Fig. 3d)—the main site for binding of the CD11b i-domain to fibrin^26^. Blockade of the fibrin amino acids 386–394 epitope with 5B8, a therapeutic mouse monoclonal antibody against the fibrin γ_377–395_ peptide^17^, inhibited the interaction between human fibrin and spike (Fig. 1k and Extended Data Fig. 3e) and suppressed spike-enhanced fibrin-induced ROS release from BMDMs (Fig. 1l). Inhibition of the fibrin–spike interaction may be influenced by the higher affinity of fibrin for 5B8 (26 nM)^17^ than for spike (390 nM), as well as additional binding sites between spike and fibrin. Stereotactic injection of fibrinogen and spike in the mouse brain increased fibrin-induced microglial reactivity (Extended Data Fig. 3f). Overall, these results reveal a role for fibrinogen as a SARS-CoV-2 spike-binding protein accelerating the formation of abnormal clots with increased inflammatory activity.

## Fibrin drives inflammation

**Fig. 2:**
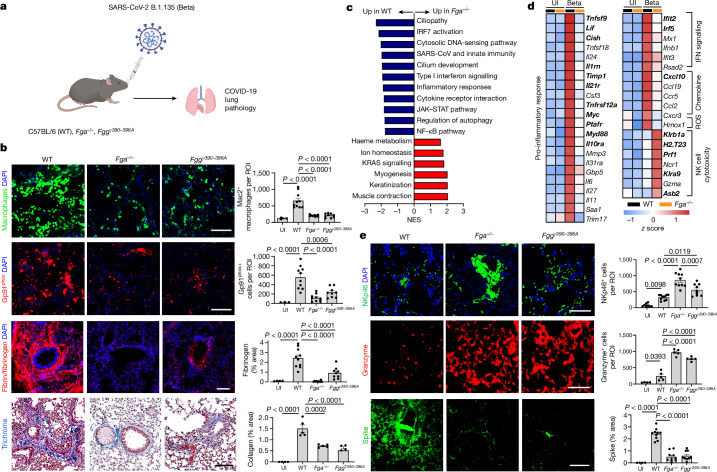
Fibrin drives lung pathology after SARS-CoV-2 infection. **a**, Lung pathology of Beta-infected WT, *Fga*^*−/−*^ and *Fgg*^*γ390–396A*^ mice. **b**, Microscopy analysis of Mac2 (macrophages) and fibrin/fibrinogen in uninfected (UI) (*n* = 4) and Beta-infected WT (*n* =10), *Fga*^*−/−*^ (*n* = 10) and *Fgg*^*γ390–396A*^ (*n* = 9) mice; gp91^phox^ in uninfected (*n* = 3) and Beta-infected WT (*n* = 10), *Fga*^*−/−*^ (*n* = 10) and *Fgg*^*γ390–396A*^ (*n* = 9) mice; and Trichrome (collagen, blue; fibrin, red) in uninfected (*n* = 4) and Beta-infected WT (*n* = 5), *Fga*^*−/−*^ (*n* = 5), *Fgg*^*γ390–396A*^ (*n* = 4) mice. Data are from mice infected in two independent experiments. **c**, Gene set enrichment analysis (GSEA) of pathways significantly altered in Beta-infected lungs of *Fga*^*−/−*^ mice compared with WT mice. NES, normalized enrichment score. **d**, Significant genes and pathways. Uninfected: *n* = 4 (WT) and *n* = 3 (*Fga*^*−/−*^) mice; Beta: *n* = 4 (WT) and *n* = 5 (*Fga*^*–/–*^) mice. **e**, Microscopy analysis of NKp46, granzyme and spike in lung after infection. NKp46: uninfected, *n* = 8 (WT); infected, *n* = 10 (WT), *n* = 10 (*Fga*^*−/−*^) and *n* = 9 (*Fgg*^*γ390–396A*^) mice; granzyme: uninfected, *n* = 4 (WT); infected, *n* = 5 mice per group; spike: uninfected, *n* = 4 (WT); infected: *n* = 10 (WT), *n* = 10 (*Fga*^*−/−*^) and *n* = 9 (*Fgg*^*γ390–396A*^) mice. Statistical analysis was performed using one-way ANOVA with Tukey’s multiple-comparison test (**b** and **e**) and two-sided quasi-likelihood *F*-test implemented in edgeR (**d**). In **d**, bold font indicates adjusted *P* < 0.05 (Benjamini–Hochberg). Each lane represents the average scaled *z*-score for each genotype. Data are mean ± s.e.m. Scale bars, 100 μm (**b** and **e**). The diagram in **a** was created with BioRender. Source Data

**Extended Data Fig. 4:**
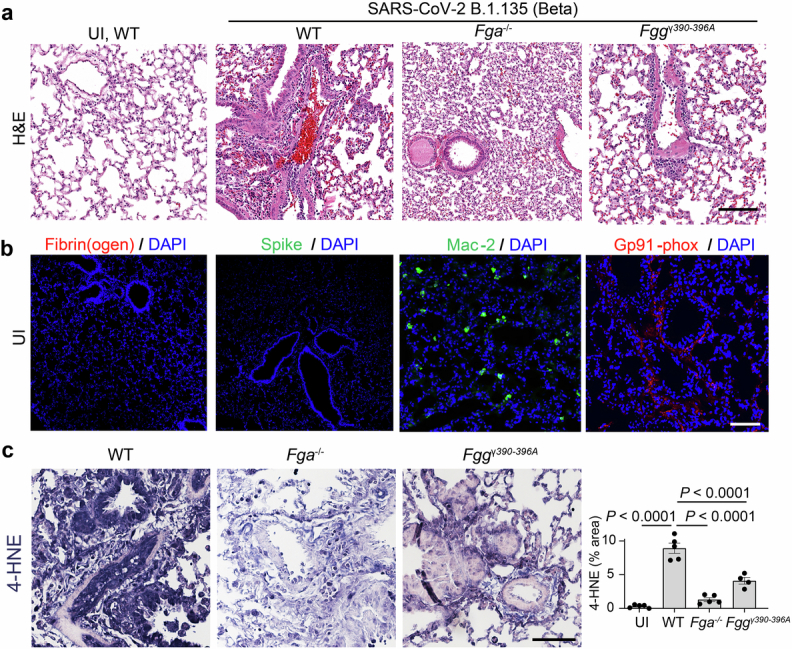
Lung microscopy from SARS-CoV-2 infection. **a**, Microscopy of hematoxylin and eosin staining from lung of WT, *Fga*^*–/–*^ and *Fgg*^*γ390–396A*^ mice after Beta infection or uninfected (UI) WT mice. Representative images from UI *n* = 4; Infected, WT *n* = 5, *Fga*^*–/–*^ *n* = 5 and *Fgg*^*γ390–396A*^ *n* = 4 mice. Scale bar, 200 μm **b**, Microscopy of fibrin(ogen) (red), Spike (green), Mac-2 (green), and gp91-phox (red) immunoreactivity in the lung from UI mice. Nuclei are stained with DAPI (blue). Scale bar, 70 μm. Representative images from *n* = 3 mice per group. **c**, Microscopy for 4-HNE in lung from UI *n* = 5 and Beta-infected *n* = 5 WT, *n* = 5 *Fga*^*–/–*^, *n* = 4 *Fgg*^*γ390–396A*^ mice. One-way ANOVA with Tukey’s multiple comparisons test. All data are mean ± s.e.m. Scale bars, 100 μm. Source Data

Conversion of fibrinogen to fibrin exposes its cryptic inflammatory γ_377–395_ epitope^26^. Genetic or pharmacological targeting of this epitope has potent therapeutic effects in autoimmune and inflammatory diseases^15,17–21^. WT, fibrinogen-deficient (*Fga*^*–/–*^) and *Fgg*^*γ390–396A*^ mice, which express mutant fibrinogen that retains normal clotting function but lacks the γ_390–396_ motif for binding to the receptor CD11b–CD18, were infected intranasally with a SARS-CoV-2 Beta variant that is naturally mouse adapted (Fig. 2a). In WT mice, infection induced macrophage infiltration and alveolar haemorrhage, and these were reduced in *Fga*^*–/–*^ and *Fgg*^*γ390–396A*^ mice (Fig. 2b and Extended Data Fig. 4a,b). Fibrin induces oxidative stress through CD11b–CD18-mediated activation of nicotinamide adenine dinucleotide phosphate (NADPH) oxidase^17,20,21^, which is linked to severe disease and thrombotic events in patients with COVID-19^27^. *Fga*^*–/–*^ and *Fgg*^*γ390–396A*^ mice had significantly less gp91^phox^ NADPH oxidase subunit and less of the oxidative stress marker 4-hydroxynonenal in the lungs after infection than did the control mice (Fig. 2b and Extended Data Fig. 4b,c). Collagen deposition in severe COVID-19 cases is linked to progressive fibrotic lung disease^28^. Collagen accumulation was significantly reduced in the lungs of infected *Fga*^*–/–*^ mice and *Fgg*^*γ390–396A*^ mice compared with WT (Fig. 2b). Fibrin deposits were absent in infected *Fga*^*–/–*^ mice, as expected, and decreased in *Fgg*^*γ390–396A*^ mice (Fig. 2b). Overall, these results suggest that fibrin signalling through CD11b–CD18 induces inflammatory cell infiltration, oxidative stress and fibrosis in SARS-CoV-2 infection, with implications for long-term complications seen in COVID-19.

**Extended Data Fig. 5:**
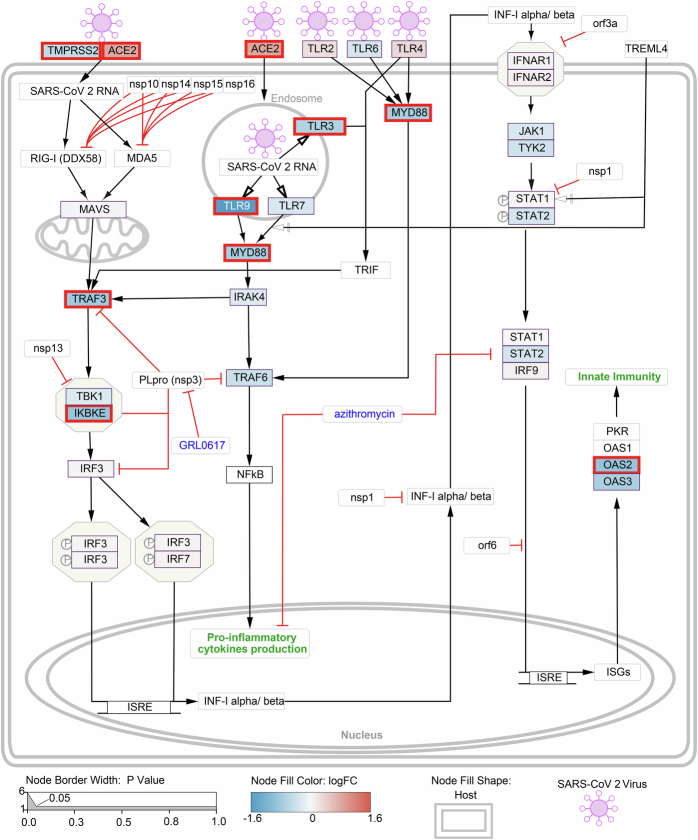
Suppression of the human type I interferon network in *Fga*^–*/*–^ mice after SARS-CoV-2 infection. Overlay of the differentially expressed genes in the lung from Beta-infected *Fga*^*–/–*^mice on the “Type I interferon induction and signalling during SARS-CoV-2 infection - Homo sapiens” pathway. Gene nodes are coloured with red-blue gradient to indicate degree of log2 fold change in gene expression between WT and *Fga*^–*/*–^ mice. Red borders indicate statistical significance of unadjusted *P* < 0.05 calculated by two-sided quasi-likelihood F-test implemented in edgeR. https://new.wikipathways.org/pathways/WP4868.html.

**Extended Data Fig. 6:**
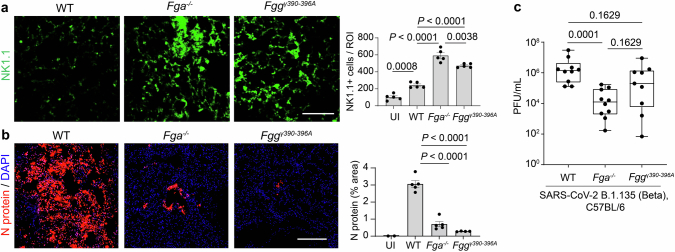
Lung pathology and viral titers after SARS-CoV-2 infection. **a**, Microscopy of NK1.1 in lung after Beta-infection at 3 d.p.i. *n* = 5 mice per group. **b**, Microscopy of N protein in lung after infection at 3 d.p.i; Infected, *n* =5 WT, *n* = 5 *Fga*^*–/–*^, *n* = 4 *Fgg*^*γ390-396A*^ mice. **c**, Box-and-whisker plots showing the number of PFUs propagated from lung homogenates of infected animals on Vero cells. PFU/ml from lung homogenates of Beta-infected *n* = 10 WT, *n* = 10 *Fga*^–*/*–^ and *n* = 9 *Fgg*^*γ390–396A*^ mice at 3 d.p.i. *P* values shown by Welch two-sample two-sided t-test followed by multiple correction testing using the Holm procedure. Box indicates the interquartile range (IQR) and whiskers denote the 1.5 × IQR. **a, b**, One-way ANOVA with Tukey’s multiple comparisons test. All data are mean ± s.e.m. Scale bars, 100 μm. Source Data

We next assessed the effects of fibrinogen on the lung transcriptome after COVID-19. Fibrinogen deficiency reduced the expression of genes of inflammatory pathways, such as SARS coronavirus and innate immunity (*Ifit2*, *Ifit3b*, *Irf5*, *Myd88*, *Cxcl10*, *Tnfsf9*, *Il1rn* and *Lif*); regulation of type I interferon (IFN) signalling (*Ifit2*, *Ifit3b* and *Irf5*); and the JAK–STAT pathway and NF-κB pathway (Fig. 2c,d and Supplementary Tables 4 and 5). The type I IFN response is elevated during active infection and persists as a biomarker for long COVID^7,14^. Overlay of gene expression data with the human type I IFN induction and signalling during SARS-CoV-2 infection pathway showed a 73% reduction in type I IFN-regulating genes in infected *Fga*^*−/−*^ mice (Extended Data Fig. 5). Indeed, expression of the type I IFN-induced gene *Cxcl10*, which encodes a key inflammatory cell chemoattractant that is induced by fibrin and is associated with cytokine storm and severe COVID-19^21,29^, was markedly reduced in infected *Fga*^*−/*^^−^ lungs compared with in the controls (Fig. 2d). By contrast, expression of the natural killer (NK) cell-expressed surface antigen-encoding genes *Klrb1a* and *Klra9* and the cytotoxic gene *Prf1* was increased in infected *Fga*^*−/*^^−^ mice compared with infected controls (Fig. 2d). Accordingly, NK1.1 expressed in NK cells, NKT cells and ILC1 cells, as well as NKp46 and granzyme, were upregulated in the lungs of infected *Fga*^*−/−*^ and *Fgg*^*γ390–396A*^ mice (Fig. 2e and Extended Data Fig. 6a), suggesting a role for fibrin as a regulator of NK cells in infection.

Reduced NK cell recruitment and activation impairs virus elimination and has been linked to poor outcomes in COVID-19^30^. Spike and N proteins were reduced in *Fga*^*−/*^^−^ and *Fgg*^*γ390–396A*^ mice compared with WT mice (Fig. 2e and Extended Data Fig. 6b). In plaque-forming assays, virus levels were reduced in lung lysates of *Fga*^*−/−*^ mice (Extended Data Fig. 6c), suggesting that the effects of fibrinogen could be attributed to regulation of immune pathways or lower levels of the virus. Although there was a trend for reduced viral titres in lung lysates of *Fgg*^*γ390–396A*^, the titres were too variable to be statistically significant (Extended Data Fig. 6c). A robust increase in NK cells coupled with decreased viral production in the lungs after fibrin depletion or inhibition of fibrin’s interaction with the receptor CD11b–CD18 during SARS-CoV-2 infection is consistent with increased activation of CD11b-deficient NK cells during tumour surveillance^31^.

## Fibrin suppresses NK cells

**Fig. 3:**
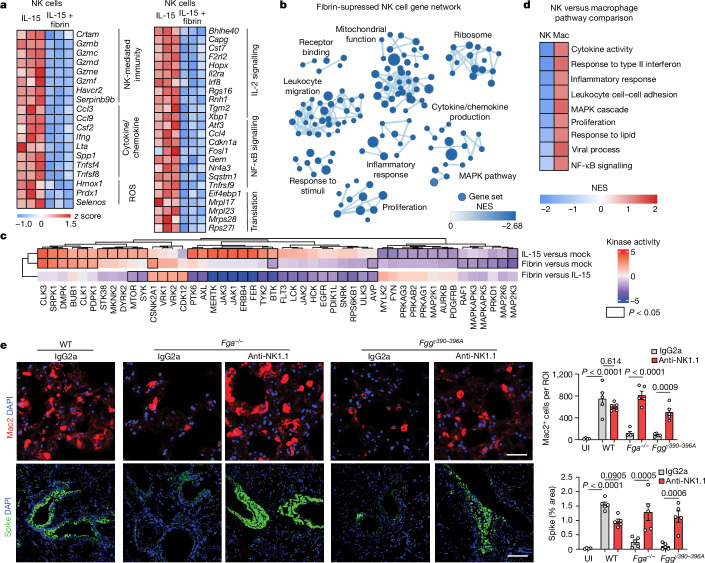
Fibrin suppresses NK cells and promotes SARS-CoV-2 infection. **a**, Heat map of selected genes and pathways from bulk RNA-seq analysis of primary mouse NK cells stimulated with fibrin for 4 days in vitro. *n* = 3 mice. Each lane represents the normalized scaled expression (*z* score) from each individual mouse (Methods). **b**, Fibrin-suppressed GO term networks from bulk RNA-seq analysis of primary mouse NK cells. Each circle represents one significantly altered pathway. NES, normalized enrichment score. **c**, Kinase activities inferred as a *z* score of phosphorylated substrates from global MS phosphoproteomics analysis of NK cells isolated from PBMCs unstimulated (mock) or treated with fibrin or IL-15 for 1 h. The colours indicate an increase (red) or decrease (blue) in kinase activity. The black bounding boxes indicate a significant shift in kinase-specific substrate regulation. Statistical analysis was performed using a two-tailed *z*-test (unadjusted *P* < 0.05) based on the log_2_-transformed fold changes between *n* = 8,054 phosphorylation sites derived from 2 (mock), 3 (fibrin) and 2 (IL-15) biologically independent experiments (Methods). **d**, The NES of selected pathways from GSEA of fibrin-induced genes in NK cells (shown in **b**) and macrophages (mac.; scRNA-seq data from a previous study^21^). **e**, Microscopy analysis of Mac2 and spike in the lungs of Beta-infected WT, *Fga*^*–/–*^ and *Fgg*^*γ390–396A*^ mice given intraperitoneal injection of anti-NK1.1 or IgG2a at a dose of 8 mg per kg body weight. Nuclei were stained with DAPI (blue). Scale bars, 50 μm (Mac2) and 200 μm (spike). Uninfected: *n* = 4 WT mice; Beta infected: *n* = 5 mice per group. Statistical analysis was performed using two-way ANOVA with Tukey’s multiple-comparison test. Data are mean ± s.e.m. Source Data

**Extended Data Fig. 7:**
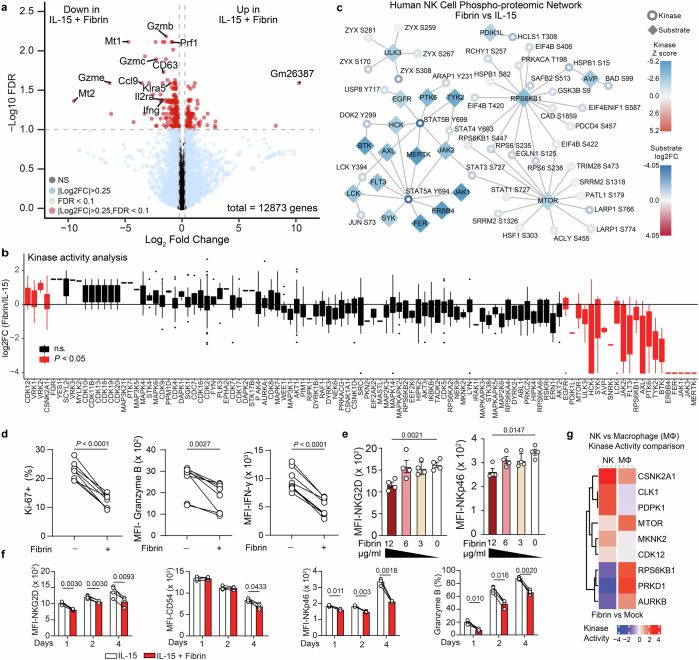
NK cell responses to fibrin stimulation. **a**, Volcano plots of DEGs from bulk RNA-seq of fibrin-stimulated mouse primary NK cells for four days. *n* = 3 mice per group. The cutoffs were Log2 fold change > 0.25, adjusted *P* (FDR) < 0.1 by two-sided quasi-likelihood F-test in edgeR with the Benjamini-Hochberg method. **b**, Differential phosphorylation site intensities between Fibrin- and IL-15-treated NK cells isolated from PBMCs. Phosphorylation sites grouped based on upstream kinases annotated in the OmniPath network database of kinase-substrate relationships. Red boxes indicate a significant shift in kinase-specific substrate regulation. Unadjusted *P* < 0.05 calculated from log2 fold changes of *n* = 8,054 phosphorylation sites between conditions derived from 2 (mock), 3 (fibrin) and 2 (IL-15) biologically independent experiments (two-tailed z-test, Methods). Box indicates the interquartile range (IQR) and whiskers denote the 1.5 × IQR. **c**, Downregulated network of phosphoproteomic interaction in fibrin-treated human NK cells for 1 h compared to IL-15. Colour of kinase represents z-score of kinase activity and colour of substrates represents log2FC (Methods). **d**, Flow cytometry of mouse NK cells fibrin-stimulated for four days. Ki-67 (%), granzyme B (MFI) and IFN-γ (MFI), *n* = 8 mice per group. Two-tailed paired Student’s t-test. **e**, Flow cytometry of fibrin-stimulated mouse NK cells for four days at concentrations indicated. NKp46 (MFI), NKG2D (MFI). *n* = 4 mice per group. One-way ANOVA with post-hoc analysis by Sidak’s multiple comparisons. Data are mean ± s.e.m. **f**, Flow cytometry of NK cells fibrin-stimulated for 1, 2, and 4 days. NKG2D (MFI), CD54 (MFI), NKp46 (MFI), and granzyme B (%). *n* = 4 mice per group. Two-tailed paired Student’s t-test. **g**, Kinase activities inferred from global mass spectrometry phosphoproteomics of NK cells and macrophages (data from Mendiola et al.^21^) unstimulated (Mock) or Fibrin-treated for 1 h. Gating strategy is shown in Supplementary Fig. 2. Source Data

To determine the mechanism of fibrin-induced NK cell suppression, we first performed bulk RNA-sequencing (RNA-seq) analysis of fibrin-stimulated primary mouse NK cells, and identified 277 downregulated genes and 76 upregulated genes (Extended Data Fig. 7a and Supplementary Table 6). Fibrin suppressed genes encoding molecules that control NK cell-mediated immunity (*Gzmb*, *Gzmc* and *Crtam*), cytokines and chemokines (*Ccl3*, *Ifng* and *Csf2*), the response to ROS (*Hmox1*, *Prdx1* and *Selenos*), IL-2 signalling (*Bhlhe40*, *Cst7* and *Il2ra*), NF-κB signalling (*Ccl4*, *Nr4a3* and *Tnfrsf9*) and translation (*Eif4ebp1*, *Mrpl17* and *Mrpl23*) (Fig. 3a and Supplementary Table 6). Fibrin markedly suppressed a network of pathways, including mitochondrial function, leukocyte migration, cytokine/chemokine production, inflammatory response, proliferation and MAPK (Fig. 3b and Supplementary Table 7). Using quantitative mass spectrometry (MS) phosphoproteomics and kinase activity analysis^32^, we globally characterized the dynamics of protein phosphorylation and kinase–substrate relationships in human NK cells in response to fibrin or IL-15 (Fig. 3c and Supplementary Tables 8–10). Fibrin downregulated the JAK–STAT pathway compared with IL-15, as well as multiple targets of the p38 MAP kinase (that is, MAP2K3, MAP2K6, MAPKAPK3, MAPKAPK5 and RAF1), consistent with the role of these pathways in regulating NK cell activation^33^ (Fig. 3c and Extended Data Fig. 7b). Phosphoproteomic network analysis revealed that, compared with IL-15, fibrin reduced the induction of JAK–STAT5, MTOR–S6K (also known as RPS6KB1) and LCK pathways (Extended Data Fig. 7c), which are essential for the effector functions, energy metabolism and survival of NK cells in COVID-19^33^. Furthermore, fibrin reduced surface expression of NK cell activation markers (NKp46, NKG2d, CD54), cell proliferation and production of IFNγ and granzyme B (Extended Data Fig. 7d–f). In contrast to its effects in primary macrophages and microglia^21^, in NK cells, fibrin suppressed cytokine activities, IFN response, inflammation, MAPK signalling, proliferation, response to lipid, viral process and NF-κB signalling (Fig. 3d). Indeed, comparison of kinase signalling responses between fibrin-treated NK cells (this study) and fibrin-treated macrophages^21^ revealed that fibrin differentially regulated signalling pathways in the two cell types (Extended Data Fig. 7g).

**Extended Data Fig. 8:**
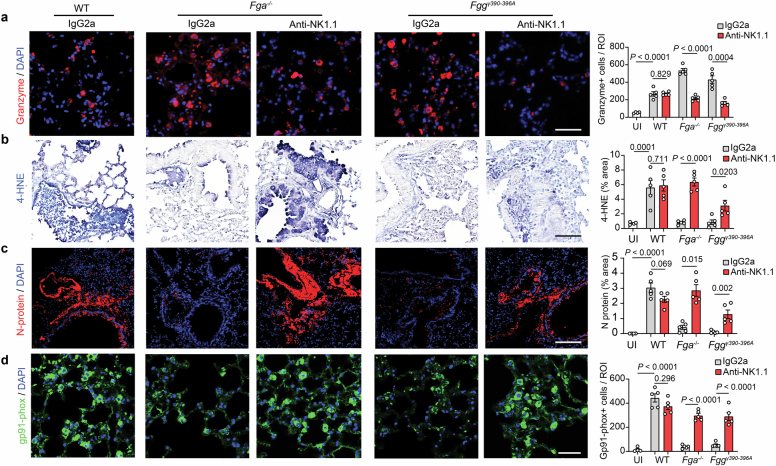
NK cell depletion in vivo. **a-d**, Microscopy of granzyme, 4-HNE, N protein and gp91-phox in lung from Beta-infected WT, *Fga*^*–/–*^, and *Fgg*^*γ390-396A*^ mice given anti-NK1.1 or IgG2a *n* = 5 mice per group. Uninfected (UI), *n* = 4 mice. Scale bars, 50 μm (a, d), 200 μm (b, c). **a-c**, Welch two-sample t-test (two-sided) followed by multiple correction testing using the Holm procedure. Unadjusted *P* values (**b**). **d**, Two-way ANOVA with Tukey multiple comparison correction. All data are mean ± s.e.m. Source Data

We next tested whether the pathogenic effects of fibrinogen in COVID-19 depend on its inhibitor effects on NK cells. We infected WT, *Fga*^*−/−*^ and *Fgg*^*γ390–396A*^ mice with SARS-CoV-2 Beta after NK cell depletion with anti-NK1.1 antibody (Supplementary Table 11). Depletion of NK1.1^+^ cells abolished the protection provided by fibrinogen depletion indicated by increased macrophages, oxidative stress, N protein and spike in *Fga*^*−/−*^ and *Fgg*^*γ390–396A*^ lungs to WT levels (Fig. 3e and Extended Data Fig. 8). These findings indicate that fibrinogen is required for SARS-CoV-2 infection in the lung and pulmonary lesion formation through inflammatory activation and suppression of viral clearance involving NK cells.

## Infection-independent fibrin functions

**Fig. 4:**
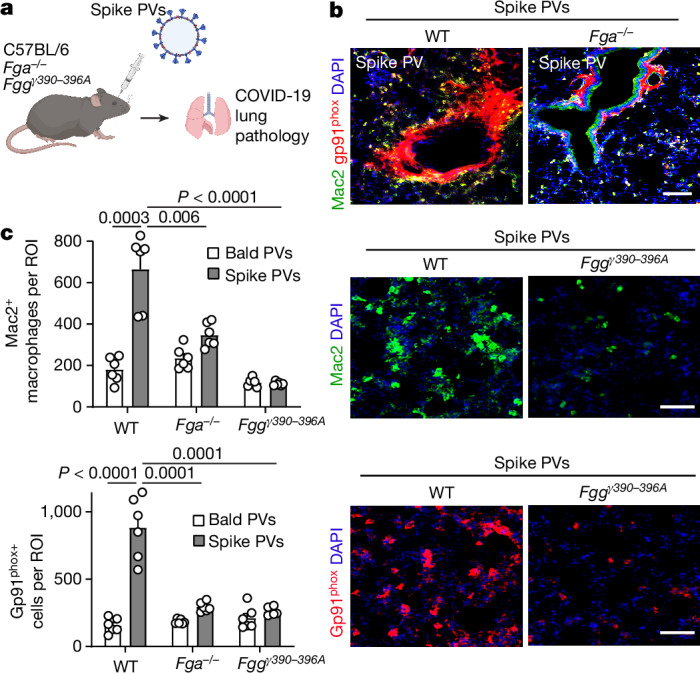
Fibrin drives infection-independent SARS-CoV-2 pathology. **a**, Lung pathology from spike PV i.v. administration in WT, *Fga*^*−/−*^ and *Fgg*^*γ390–396A*^ mice. The diagram was created using BioRender. **b**,**c** Mac2 and gp91^phox^ microscopy and quantification in lungs of WT, *Fga*^*–/–*^ and *Fgg*^*γ390–396A*^ mice after bald or spike PV administration. *n* = 6 mice per group. Statistical analysis was performed using two-tailed Welch two-sample *t*-tests followed by multiple-correction testing using the Holm procedure. Data are mean ± s.e.m. Scale bars, 50 µm (**b** and **c**). Source Data

**Extended Data Fig. 9:**
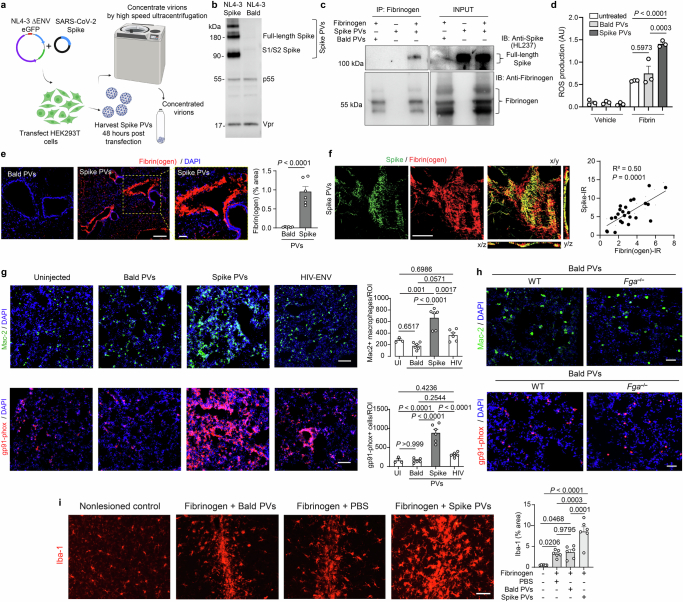
Production of PVs and in vivo characterization. **a**, Spike PV production (Methods). **b**, Immunoblot of Spike expression in PVs blotted with anti-Spike, anti-p24 Gag (detecting p55) and anti-Vpr. Spike PVs expressed S1, S2, cleaved S1 and Spike multimeric forms. PVs express comparable levels of the proviral backbone indicated by HIV Env VPs (Vpr). **c**, Fibrinogen immunoprecipitation with PVs blotted with anti-Spike or anti-fibrinogen. **d**, ROS production in fibrin-stimulated BMDMs (24 h) with PVs. *n* = 3 biologically independent experiments. **e**, Fibrin(ogen) from lungs of *n* = 6 WT mice per group 24 h after PV injection. Scale bars, 200 µm and 50 µm (inset). Welch two-sample t-test (two-tailed) followed by Holm multiple correction testing. **f**. Confocal microscopy of Spike (green) and fibrin(ogen) (red) in lung 24 h after Spike PVs injection; orthogonal views of the y/z and x/z planes show the localization of fibrinogen and Spike. Scale bar, 50 μm. Scatter plot shows correlation of fibrinogen and Spike in *n* = 24 images from three mice, Pearson correlation (Methods). **g**, gp91-phox (red) and Mac-2 (green) in lungs 24 h after injection of *n* = 6 mice (Bald, Spike or HIV-ENV PVs) and *n* = 3 uninjected controls (UI). Scale bars, 100 μm. **h**, Mac-2 (green) and gp91-phox (red) in lungs from WT and *Fga*^*–/–*^ mice after Bald PVs injection. Scale bar, 70 μm. Representative images from *n* = 6 mice per group. Quantification in Fig. 4c. **i**, Iba-1 in corpus callosum after stereotactic co-injection of fibrinogen with PBS, Bald PVs or Spike PVs. Scale bar, 50 µm. *n* = 6 mice per group. Representative immunoblots from two (**b**) or three (**c**) biologically independent experiments. **d, g, i**, One-way ANOVA with Tukey’s multiple comparisons test. All data are mean ± s.e.m. For gel source data, see Supplementary Fig. 1. Source Data

Persistent circulating SARS-CoV-2 spike has been reported in long COVID^34^. We hypothesized that the interplay between fibrin and spike might regulate thromboinflammation in COVID-19 beyond active infection. We generated HIV virions pseudotyped with trimeric spike (spike PVs) that are unable to engage mouse ACE2 receptors (Extended Data Fig. 9a,b). Similar to recombinant spike (Fig. 1), spike PVs co-immunoprecipitated with fibrinogen and increased fibrin-induced oxidative stress in BMDMs (Extended Data Fig. 9c,d). Spike PVs given by i.v. injection into WT mice induced extensive fibrin deposition in the lungs (Fig. 4a and Extended Data Fig. 9e,f). In WT mice, spike PVs activated macrophages and increased expression of gp91^phox^ in the lungs, indicating oxidative stress (Extended Data Fig. 9g). By contrast, control bald PVs or PVs expressing the Env protein from the HIV-1 (HIV-1 PVs) did not induce these effects (Extended Data Fig. 9g), suggesting that lung pathology was specific for spike. *Fga*^*−/−*^ and *Fgg*^*γ390–396A*^ mice had reduced macrophage activation and oxidative stress in the lungs after spike PV administration (Fig. 4b,c and Extended Data Fig. 9h). In a mouse model of fibrinogen-induced encephalomyelitis^35^, co-injection of spike PVs increased fibrin-induced microglial reactivity (Extended Data Fig. 9i), suggesting that spike enhances the inflammatory function of fibrin in vivo. These results suggest a fibrin-dependent mechanism that elicits inflammatory and oxidative stress responses in the presence of spike in the absence of active infection, which could therefore have a role in long COVID. Notably, we do not believe that this mechanism is related to the rare clotting complications observed with adenovirus based COVID vaccines because the production of anti-PF4 autoantibodies and ensuing drop in platelet counts are triggered by the vector rather than spike^36^. In general, COVID-19 RNA vaccines lead to small amounts of spike protein accumulating locally and within draining lymph nodes where the immune response is initiated and the protein is eliminated^37^. Consistent with the safety of the spike mRNA vaccines, mRNA vaccines prevent post-COVID-19 thromboembolic complications^38^ and a cohort study in 99 million COVID-vaccinated individuals showed no safety signals for haematological conditions^39^.

## Fibrin-targeting antibody in SARS-CoV-2

**Fig. 5:**
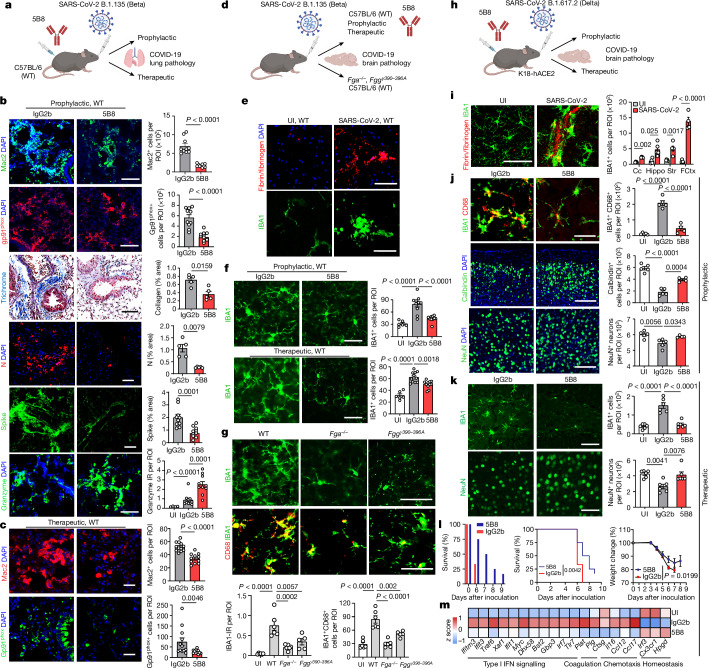
Anti-fibrin antibody provides protection against SARS-CoV-2. **a**, Beta infection of 5B8-treated WT mice. **b**,**c**, Lung pathology in WT mice prophylactically treated with 5B8 or IgG2b (*n* = 5 (Trichrome, N protein); *n* = 10 (Mac2, gp91^phox^, spike, granzyme)) at 3 d.p.i. (**b**) or therapeutically treated with 5B8 (*n* = 11) or IgG2b (*n* = 12) (Mac2 and gp91^phox^) at 7 d.p.i. (**c**). **d**, Beta infection of WT, *Fga*^−/−^ and *Fgg*^*γ390–396A*^ mice or 5B8-treated WT mice at 7 d.p.i. **e**, Fibrinogen and IBA1 in the cortex, representative of four Beta-infected WT mice. **f**, IBA1 in the hippocampus. UI: *n* = 6 mice; Beta infected, prophylactic: *n* = 10 (prophylactic 5B8 or IgG2b) mice per group; Beta infected, therapeutic: *n* = 12 (IgG2b) and *n* = 11 (5B8) mice. **g**, IBA1 and CD68 in the hippocampus. Uninfected: *n* = 6 WT mice; Beta infected, *n* = 6 (WT), *n* = 6 (*Fga*^*−/−*^) or *n* = 5 (*Fgg*^*γ390–396A*^) mice. **h**, Delta infection of 5B8-treated K18-hACE2 mice. **i**, Fibrinogen and IBA1 in various brain regions of uninfected and Delta-infected mice at 3 d.p.i. Uninfected: *n* = 4 (hippocampus (Hippo)) and *n* = 5 (corpus callosum (Cc), striatum (Str) and frontal cortex (FCtx)) mice; Delta infected: *n* = 4 (frontal cortex) and *n* = 5 (hippocampus, corpus callosum, striatum) mice. **j**,**k**, IBA1, CD68, calbindin and NeuN in the cortex (**j**) and hippocampus (**k**). Uninfected: *n* = 5 mice; Delta infected, prophylactic, 3 d.p.i.: *n* = 5 (IgG2b) or *n* = 4 (5B8) mice; Delta infected, therapeutic, 9 d.p.i.: *n* = 6 mice per group. **l**, Mouse survival and weight. *n* = 12 mice per group (therapeutic, 5B8 or IgG2b, Delta infected). Statistical analysis was performed using log-rank tests (survival) and a mixed-effects model (weight). **m**, Significantly altered genes in the hippocampus of Delta-infected mice given 5B8 or IgG2b. *n* = 6 mice per group. Statistical analysis was performed using two-sided unpaired *t*-tests (unadjusted *P* < 0.05; Methods). For **a**–**f** and **h**–**m**, 5B8 or IgG2b was given intraperitoneally at a dose of 30 mg per kg body weight, prophylactically (at 0 d.p.i.) or therapeutically (at 1 d.p.i.). Statistical analysis was performed using two-tailed Mann–Whitney *U*-tests (**b** (all except for granzyme) and **c**), two-tailed Welch *t*-tests with Holm multiple-comparison correction (**b** (granzyme) and **i**) and one-way ANOVA Tukey’s multiple-comparison test (**f**, **g**, **j** and **k**). Data are mean ± s.e.m. Scale bars, 100 μm (**b**, **c**, **e**, **j** and **i**) or 50 μm (**f**, **g** and **k**). The diagrams in **a**, **d** and **h** were created using BioRender. Source Data

**Extended Data Fig. 10:**
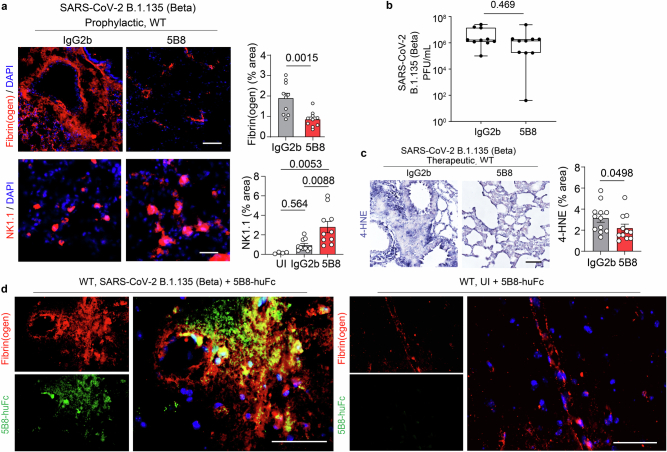
5B8 target engagement in the brain and effects on lung pathology in Beta infection. **a**, Microscopy of fibrinogen and NK1.1 in lungs from prophylactic 5B8- and IgG2b-treated Beta-infected WT mice 3 d.p.i. Scale bars, 100 μm. Infected, *n* = 10 mice per group; uninfected (UI), *n* = 4 mice. Two-tailed Mann-Whitney test (fibrin(ogen)), one-way ANOVA with Tukey multiple comparisons (NK1.1). **b**, Box-and-whisker plots showing the number of PFUs propagated from lung homogenates of infected animals on Vero cells at 3 d.p.i. PFU/ml from lung homogenates of Beta-infected WT mice given prophylactically 5B8 or IgG2b. *n* = 10 per group. Two-tailed Mann-Whitney. Box indicates the interquartile range (IQR) and whiskers denote the 1.5 × IQR. **c**, Microscopy of 4-HNE in lungs from therapeutic 5B8- and IgG2b-treated WT mice after Beta infection at 7 d.p.i. Scale bars, 100 μm. *n*  = 12 (IgG2b) or *n* = 11 (5B8). Two-tailed Mann-Whitney test. **d**, Microscopy of brain sections from Beta-infected WT (left) and UI control (right) given i.p. injection of 30 mg/kg 5B8-huFc showing the spatial co-localization (yellow) between 5B8-huFc detected with FITC-human IgG (green), and fibrin deposition detected with antibody to fibrin(ogen) (red) at 7 d.p.i. The 5B8-huFc antibody was used in target engagement studies to enable in vivo detection in the mouse by human FITC-IgG. Scale bars, 80 μm. Data are representative of *n* = 3 mice. All data are mean ± s.e.m. Source Data

Neutralizing fibrin toxicity is an attractive therapeutic strategy for neuroprotection and selective suppression of pathogenic inflammation^17,40^. The monoclonal antibody 5B8 targeting the fibrin inflammatory domain γ_377–395_ provides protection from autoimmune- and amyloid-driven neurodegeneration without adverse effects on haemostasis^17^. We tested the effects of 5B8 after i.n. infection with two different variants of SARS-CoV-2 in models with and without neuroinvasion, as well as in the spike PV non-infectious model (Supplementary Tables 12 and 13). Mice were infected with 10^4^ plaque-forming units (PFU) and 10^3^ PFU for 3 and 7 days post infection (d.p.i.), respectively, for optimal survival and pathological alterations. In WT mice infected with SARS-CoV-2 Beta, prophylactic administration of 5B8 reduced macrophage activation, oxidative stress, collagen accumulation, fibrin deposition and viral spike and N protein expression, while increasing NK cell responses in the lungs compared with the isotype IgG2b-treated controls (Fig. 5a,b and Extended Data Fig. 10a). No differences in the viral titres in the lung lysates were observed, potentially due to titre variability (Extended Data Fig. 10b). Therapeutic 5B8 administration 24 h after infection decreased macrophage activation and oxidative stress assessed at 7 d.p.i. (Fig. 5c and Extended Data Fig. 10c). 5B8 spatially correlated with fibrin-rich areas in the brain of Beta-infected WT mice (Extended Data Fig. 10d), demonstrating target engagement. These findings suggest that fibrin-targeting immunotherapy suppresses SARS-CoV-2 pathogenesis.

COVID-19 neuropathology is characterized by microglial reactivity and fibrin deposition, grey matter decrease, microhaemorrhages and small infarcts, and myelin alterations^2,3,8,9^. COVID-19 neurological symptoms and neuropathological alterations have been attributed to secondary effects of systemic SARS-CoV-2 infection, such as cytokine storm and thrombotic complications, or to direct viral infection of the brain^2,7,14,41^. We first tested the role of fibrin in the absence of brain infection using a SARS-CoV-2 Beta mouse-proficient variant that is not associated with neuroinvasion^42^ (Fig. 5d). In Beta-infected C57BL/6 mice, we found fibrin deposits in the brain at sites of microglial reactivity at 7 d.p.i. (Fig. 5e), reminiscent of neuropathologic alterations observed in patients with COVID-19, as well as infected hamsters or mice^8,9,43,44^. Prophylactic or therapeutic administration of 5B8 decreased microglial reactivity in the hippocampus after Beta infection compared with the IgG2b isotype control (Fig. 5f). Accordingly, *Fga*^*−/−*^ and *Fgg*^*γ390–396A*^ mice had reduced microglial reactivity in the hippocampus after SARS-CoV-2 Beta infection (Fig. 5g), suggesting that fibrin promotes neuroinflammation in COVID-19.

**Extended Data Fig. 11:**
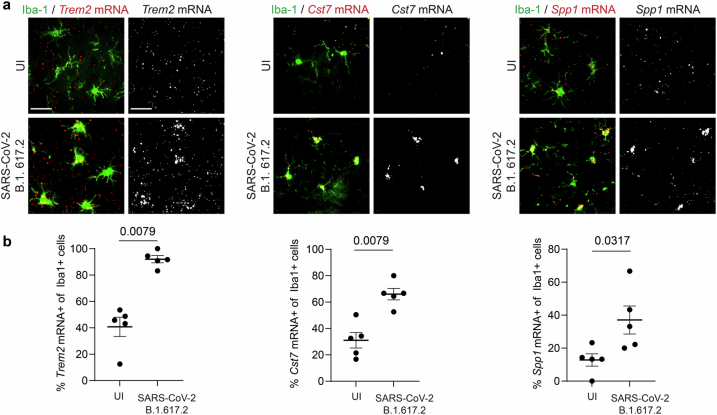
Disease-associated genes in microglia in Delta-infected mice. **a**, Representative RNAscope images of *Trem2*, *Cst7*, and *Spp1* (red) mRNA expression in Iba-1 immunoreactive microglia (green) within the hippocampus of Delta-infected K18-hACE2 mice. **b**, Quantification of Trem2, Cst7, and Spp1 mRNA expression as percentage of total Iba-1+ cells in the hippocampus. Data are from *n* = 5 mice per group. Two-tailed Mann-Whitney test. Scale bars, 20 μm. All data are mean ± s.e.m. UI, uninfected. Source Data

**Extended Data Fig. 12:**
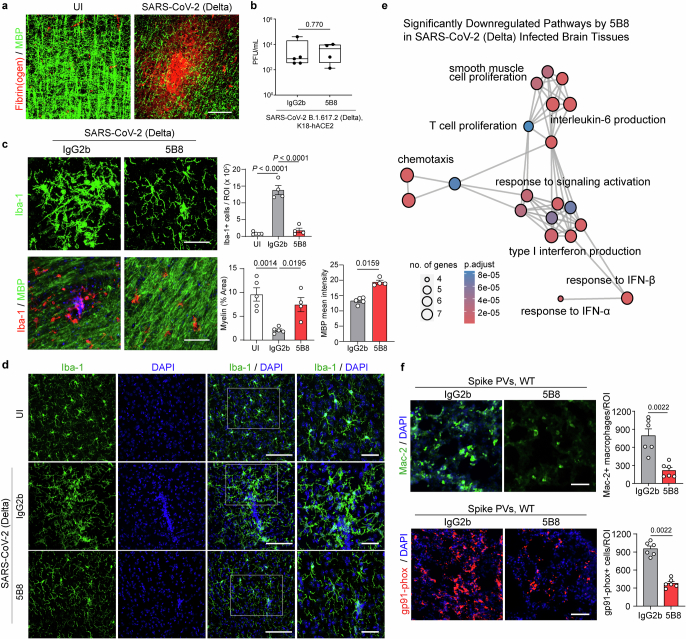
Fibrin-targeting immunotherapy protects from SARS-CoV-2 Spike neuroinflammation. **a**, Confocal microscopy of fibrin(ogen) and MBP in brains from Delta-infected K18-hACE2 mice at 3 d.p.i. Representative images from five mice per group. UI, uninfected. Delta-infected K18-hACE2 mice were given IgG2b isotype control. Scale bar, 60 μm. **b**, Box-and-whisker plots showing the number of PFUs propagated from lung homogenates of infected animals on Vero cells at 3 d.p.i. PFU/ml from lung homogenates of Delta-infected K18-hACE2 mice given prophylactically 5B8 (*n* = 4) or IgG2b (*n* = 5). Two-tailed Mann-Whitney. Box indicates the interquartile range (IQR) and whiskers denote the 1.5 × IQR. **c**, Confocal microscopy of Iba-1 and Iba-1 and MBP in brains from Delta-infected K18-hACE2 mice treated prophylactically with 5B8 or IgG2b. Scale bars, 50 μm (top) and 100 μm (bottom). UI, *n* = 5 mice; infected, 5B8 *n* = 4 mice and IgG2b *n* = 5 (MBP), *n* = 4 (Iba-1) mice. One-way ANOVA with Tukey’s multiple comparisons test or two-tailed Mann-Whitney test. **d**, Confocal microscopy of Iba-1 immunoreactivity in cortex from Delta-infected K18-hACE2 mice treated prophylactically with 5B8 or IgG2b. Representative images from five uninfected and four infected mice per group. Right-most image shows magnification of the white box. Scale bar, 100 μm; 50 μm (inset). **e**, GSEA analysis of the top 20 down-regulated pathways in brain tissues at 9 d.p.i from Delta-infected K18-ACE2 mice treated therapeutically with 5B8 versus IgG2b, starting at 1 d.p.i then every 2 days for 8 days. **f**, Microscopy of Mac-2 and gp91-phox in lungs from Spike PV-injected WT mice at 24 h given prophylactically 5B8 or IgG2b. Scale bars, 50 μm. *n* = 6 mice per group. Two-tailed Mann-Whitney test. All data are mean ± s.e.m. Source Data

We next tested the effects of fibrin immunotherapy on the K18-hACE2 mouse model of neuroinvasion using the Delta SARS-CoV-2 variant (B.1.617.2), which has been associated with risk of long COVID^45,46^ (Fig. 5h). Brains from intranasally infected K18-hACE2 mice had extensive microgliosis as reported previously^45^, associated with upregulation of disease-associated microglial markers (Fig. 5i and Extended Data Fig. 11). We found fibrin deposits at sites of microglial reactivity and decreased myelin intensity in the frontal cortex and rostral migratory stream area (Fig. 5i and Extended Data Fig. 12a). No differences in viral titres in the lung lysates were observed potentially due to titre variability (Extended Data Fig. 12b). Prophylactic 5B8 administration decreased microglial reactivity and white-matter injury compared with the IgG2b isotype control (Fig. 5j and Extended Data Fig. 12c,d). In infected mice, 5B8 reduced the loss of cortical neurons or calbindin-expressing interneurons (Fig. 5j), a feature of severe COVID-19 brain pathology associated with microglial nodules and neurovascular injury^9^. Therapeutic treatment with 5B8 at 1 d.p.i. inhibited microglial reactivity and increased neuronal survival (Fig. 5k). Therapeutic 5B8 also improved the survival rates with concomitant effects on weight loss (Fig. 5l). As K18-hACE2 mice had to be euthanized after reaching the humane end points^47^, the effects on survival could not be assessed past day 9. Transcriptomic analysis of the brains of Delta-infected K18-hACE2 mice showed that 5B8 suppressed genes encoding proinflammatory cytokines/chemokines (*Il17c*, *Ifna2*, *Il22*, *Il16*, *Cxcl10*, *Ccl12* and *Ccl17*), IFN-induced genes (*Ifit1*, *Ifit3*, *Ifi44*, *Irf7* and *Ifitm3*), and genes encoding receptors (*Tlr7*, *Il6ra*, *Il17rc*) and coagulation factors (*Plat* and *Plg*) while increasing the expression of homeostatic genes (*Cx3cr1*, *Irf3* and *Hpgd*) (Fig. 5m and Supplementary Table 14). Gene Ontology network analysis revealed that 5B8 downregulated pathways related to proliferation, IL-6 signalling, chemotaxis and response to type I IFNs (Extended Data Fig. 12e and Supplementary Table 15), consistent with human multiomic profiling of brains of individuals with COVID-19^7^. Finally, 5B8 reduced inflammation and oxidative stress in spike PV-injected mice (Extended Data Fig. 12f), suggesting that neutralizing fibrin could have a protective effect during persistent presence of spike beyond the active infection. Thus, fibrin-targeting immunotherapy provides protection from pulmonary pathology, neuroinflammation and neurodegeneration in COVID-19.

## Discussion

Although clotting complications in COVID-19 have previously been attributed primarily to systemic inflammation^14^, our findings suggest that coagulopathy in COVID-19 is not merely a consequence of inflammation, but rather serves as an apical driver of infection-induced thromboinflammation and neuropathology. Our data reveal a causal immunomodulatory role for fibrinogen in thromboinflammation and neuropathology in COVID-19. Our findings suggest that fibrin promotes neuropathological alterations either indirectly by inducing hyperinflammation through modulation of NK cells and macrophages in the infected lung or directly on microglia, owing to its parenchymal deposition in the brain after extravasation through a leaky BBB. Indeed, fibrin was sufficient to induce heightened microglia reaction in the presence of spike in the brain even in the absence of peripheral infection. Furthermore, fibrin blockade abolished neuropathology in COVID-19 animal models regardless of neuroinvasion. The finding that 5B8 anti-fibrin antibody blocks many of the pathological effects of fibrin in infected animals raises the possibility for therapeutic intervention in this thromboinflammatory pathway. This mechanism might perpetuate the hypercoagulable and proinflammatory state at sites of microvascular injury, as has been reported in patients with acute infection and long COVID^1,4^.

We show that fibrin has an immunomodulatory role promoting increased viral load and thromboinflammation in COVID-19. NK cell recruitment and activation are modulated by extracellular stimuli and interactions with monocytes and dendritic cells^33^. Through genetic loss-of-function studies, multiomics and functional assays on primary mouse and human NK cells, we show that fibrin suppresses transcriptomic and phosphoproteomic signal transduction pathways controlling NK cell cytotoxicity, proliferation and migration. Our in vitro experiments in purified NK cells stimulated with IL-15 suggest that fibrin interferes with IL-15 signalling. Thus, the effects of fibrin in vivo could be due to either regulation of IL-15 signal transduction or by limiting IFN and downstream IL-15 levels. These findings support a model in which coagulopathy functions as an extrinsic signal that may negatively regulate NK cell effector functions or recruitment through fibrin deposition. A procoagulant state leading to fibrin deposition in tissues may be particularly relevant to the impaired clearance of viral infections, where misdirected NK cells and activated macrophages contribute to disease severity. The fibrin-induced suppression of NK cells that we observed is consistent with enhanced cancer cell survival in vitro after co-culture with fibrin-stimulated NK cells^48^, suggesting a role for fibrin in other diseases with vascular damage and impaired NK cell cytotoxicity, such as cancer and autoimmune diseases^49^.

Increased BBB permeability associated with parenchymal fibrin deposition is a feature of COVID-19 neuropathology^8,9^. In the brain of some patients with COVID-19, detection of spike and viral RNA suggests potential neuroinvasion^41,45^. Our data and previous literature support that, while spike can enhance fibrin toxicity, even in the absence of spike, fibrin is deleterious in diseases such as multiple sclerosis, Alzheimer’s disease, rheumatoid arthritis, colitis and periodonditis^15,18–20^. Thus, fibrin may be deposited either together with spike when spike is present in the brain^45^ or through an open BBB after peripheral infection without neuroinvasion or spike coupling. Accordingly, the in vivo efficacy of 5B8 could depend on the inhibition of fibrin binding to spike (this study) or to its anti-inflammatory properties in brain and periphery at sites of fibrin deposition^17,40^, suggesting a dual mechanism of action for the fibrin immunotherapy in COVID-19. Given the hypercoagulable state in patients with COVID-19 with brain fog and the role of elevated plasma fibrinogen in increasing BBB permeability in mice, high plasma fibrinogen levels in COVID-19 may contribute to BBB disruption and ensuing neuropathology^5,50^. Importantly, we show that targeting fibrin is neuroprotective regardless of animal model, viral strain or neuroinvasion, suggesting a global deleterious role for fibrin in COVID-19 neuropathology.

Our study has several limitations. While we used quantification of myelin basic protein (MBP) intensity and the percentage of MBP^+^ area to demonstrate decreased myelin, future studies using electron microscopy will be required to measure demyelination. As the physiological spike concentration in the brain is not fully known, dose–response studies would be required to identify the lowest spike concentration that can enhance fibrin-induced neuroinflammation. In addition to lung, other tissues, such as heart, gut and brain, can be analysed after administration of spike PVs to test the role of fibrin in non-infectious animal models. We performed proof-of-principle in vivo studies to test the efficacy of 5B8 in three animal models of COVID-19. Further preclinical pharmacology will be necessary to evaluate the therapeutic window, dose–response of antibody and viral titres, role of mouse age and genetic background and therapeutic effects in additional species. Given the heterogenous patterns of COVID-19 neuropathology influenced by disease severity and viral strain^44^, the thromboinflammation mechanism that we described represents only one of the pleiotropic mechanisms of neuroinflammation within the spectrum of COVID-19.

Together, data from pathology, radiology and serology in patients with acute COVID-19 and long COVID^1,5,6,8–10,22,23^, as well as the genetic loss-of-function, pharmacological and transcriptomic studies in three animal models of COVID-19 (this study), establish fibrin as a key driver of inflammation and neuropathology in SARS-CoV-2 infection. Fibrin immunotherapy may represent a strategy for reducing systemic thromboinflammation and neurological manifestations of COVID-19 in both acute and long COVID. Compounded by cumulative risk of memory impairment and cognitive disorders due to breakthrough COVID-19, additional strategies are needed to provide protection against the long-term disease burden^4^. Fibrin immunotherapy may protect from cognitive symptoms associated with COVID-19, as genetic elimination of the fibrin inflammatory epitope protects Alzheimer’s disease mice from synapse loss and cognitive impairment^20^. The fibrin inflammatory epitope is not required for fibrin polymerization or platelet aggregation, and in contrast to anticoagulant therapies, it does not increase bleeding risk^15^. Accordingly, 5B8 does not affect normal clotting time in vivo, fibrin polymerization in vitro or activated partial thromboplastin time in human plasma^17^. Thus, fibrin-targeting immunotherapy may represent an approach to selectively suppress COVID-19 pathogenesis in the brain and other organs without adverse effects on normal haemostasis. A humanized affinity-matured derivative of 5B8 has entered phase 1 clinical trials in healthy individuals to assess safety and tolerability^51^. Safety trials will need to be completed for the antibody to qualify for entry into phase 2 trials to assess exploratory clinical end points. As fibrinogen plasma levels in acute COVID-19 are a predictive biomarker for cognitive impairment in long-COVID, it could be used to stratify patients as candidates for entry into phase 2 trials. Fibrin immunotherapy can be tested for its potential to reduce adverse health outcomes due to long COVID as part of a multipronged approach with prevention and vaccination measures.

## Methods

### Animals

C57BL/6 mice and K18-hACE2 mice (strain: B6.Cg-Tg(K18-ACE2)2Prlmn/J) were purchased from the Jackson Laboratory. *Fga*^*−/−*^ mice^52^ and *Fgg*^*γ390–396A*^ mice^53^ were obtained from J. Degen. Mice were housed under a 12 h–12 h light–dark cycle, 55 ± 5% relative humidity at 20 ± 2 °C with access to standard laboratory chow and water ad libitum. Both male and female mice were used. The mouse ages are indicated for each experimental procedure and were within 3 to 7 months of age. All infection experiments were performed at an AAALAC-accredited ABSL3 facility at Gladstone Institutes. All of the animal procedures were performed under the guidelines set by the Institutional Animal Care and Use Committee at the University of California, San Francisco.

### Human plasma and PBMCs

Human citrated plasma (IPLASEATNAC50ML, 1151254) was purchased from Innovative Research. Fresh PBMCs (LP,FR,MNC,2B; 3118730 and 3112992) were purchased from AllCells. All human material used in the study is commercially available and no human participants were recruited.

### SARS-CoV-2 recombinant trimeric spike protein production

The plasmid vector pCAGGS containing the SARS-CoV-2,Wuhan‐Hu‐1 ectodomain spike gene with a deletion of the polybasic cleavage site (RRAR to A), two stabilizing mutations (K986P and V987P), a C-terminal thrombin cleavage site, T4 foldon trimerization domain and a hexahistidine tag (6×His) was obtained from BEI Resources (deposited by F. Krammer)^54^. Recombinant spike was produced by transient transfection in CHO cells by Celltheon. Spike was purified by Ni^2+^-NTA affinity chromatography, eluted in phosphate-buffered saline (PBS) containing imidazole, buffer exchanged into 1× PBS and purified by size-exclusion chromatography (Superdex 200 column).

### Plasma clot formation assay

Fibrin polymerization in a plasma clot assay was measured by turbidity^17^. In brief, healthy donor citrated human plasma (Innovative Research) was diluted 1:3 in 20 mM HEPES. Recombinant spike was buffer-exchanged into 20 mM HEPES, pH 7.4, 137 mM NaCl (Amicon concentrators, 100 kDa cut-off). Equal volumes (50 µl) of plasma and buffer-exchanged spike were incubated at 25 °C for 15 min. Clotting was initiated by 0.25 U ml^−1^ thrombin (Sigma-Aldrich) and 20 mM CaCl_2_. The final concentrations were 1:12 plasma, 0.75 μM spike, 0.25 U ml^−1^ thrombin, 20 mM CaCl_2_. Turbidity was measured at 340 nm every 15 s for 30 min on the SpectraMax M5 microplate reader (Molecular Devices) using SoftMax Pro v.5.2 (Phoenix Technologies).

### SEM analysis of fibrin clots

Healthy donor citrated human plasma was diluted 1:3 in 20 mM HEPES buffer, pH 7.4; 15 μl of diluted plasma was mixed with 15 μl of recombinant spike that was buffer-exchanged into 20 mM HEPES and 137 mM NaCl (Amicon concentrators, 100 kDa cut-off) using a low concentration of NaCl to maintain spike solubility and stability. Then, 25 μl of this mixture was pipetted onto 5 mm × 5 mm silicon wafers (Ted Pella) and incubated for 15 min at 37 °C in a humidified tissue culture incubator. Then, 25 µl of a CaCl_2_ and thrombin solution in 20 mM HEPES was added in the centre of the wafer and allowed to polymerize at 25 °C for 2 h. The final concentrations were as follows: plasma 1:12, 0.9 μM spike, 0.25 U ml^−1^ thrombin, 20 mM CaCl_2_. Buffer was used instead of spike for vehicle control. Clots on wafers were placed onto ice, washed twice for 10 min each with ice-cold EM-grade 0.1 M cacodylate buffer, pH 7.4, and fixed with cold EM-grade 2% glutaraldehyde (Electron Microscopy Sciences). The samples were rinsed three times for 5 min in Millipore-filtered, double-distilled water; dehydrated in an ethanol series (20%, 50%, 70%, 90%, 100%, 100% for 2 min each); and critical-point dried with CO_2_. The samples were sputter coated with a thin layer of gold–palladium and imaged on the Zeiss Merlin field-emission SEM at 3.0 keV and a secondary electron detector.

Images at a magnification of ×4,000 were captured across the sample, then were converted to 8-bit using NIH ImageJ (v.1.50). After pixel to μm scaling, each image was cropped into two or three FOVs (8 × 8 μm) using NIH DiameterJ as described previously^55^. The surface plot plug-in in ImageJ generated topographical maps of SEM images. In brief, the best segmentation algorithm was pre-selected based on side-by-side comparison of images before quantification. The Mixed Segmentation (M1 through M3) built in DiameterJ Segment provided the most accurate representation of the fibres to be quantified. The same segmentation method and variant was used across all test conditions and images. Each segmented image was manually edited using ImageJ to ensure complete representation of segmented fibres. The edited images were batch processed using DiameterJ 1-108 (orientation analysis not selected). Fibre radius and intersection densities were collated from each batch. Data from 8–10 FOVs per sample were used for group analysis. Fibre radius distribution in Fig. 1f was calculated using FOVs from all images collected to assess the distribution across the dataset. Fibre radius proportion was statistically analysed based on three biologically independent experiments in Fig. 1f and the quantification and statistical analysis of the individual images from these experiments is shown in Extended Data Fig. 3c. Samples with collapsed fibres due to potential SEM critical-point drying technical artifacts were excluded from further analysis.

For quantification of the fibrin clots by SEM, at each radius, the difference in log-transformed odds ratio of detecting fibres (among all the views in a given image) with the chosen radius under spike versus control conditions was estimated across all images. The log-transformed odds ratio at each radius was estimated using generalized linear mixed-effects models, with the family argument set to binomial and implemented in glmer function in the lme4 (v.1.1-27) package in R^56^, in which the image source for the observations is modelled as a random effect. The *P* values were corrected for multiple testing using the Holm procedure^57^. In Fig. 1f, the *P* value represents the significance at each radius across the range of the radii between the two vertical dotted lines. The solid lines represent the best loess fit curves with span parameter set to 0.45.

### Fibrinogen- and fibrin-coated ELISA plates

Fibrinogen- and fibrin-coated plates were prepared as described previously^17^. In brief, human plasminogen-free fibrinogen (EMD Millipore) was further diluted to 25 µg ml^−1^ by adding 20 mM HEPES buffer, pH 7.4 for coating fibrinogen plates or 20 mM HEPES buffer pH 7.4 with 1 U ml^−1^ thrombin (Sigma-Aldrich) and 7 mM CaCl_2_ for fibrin-coated plates. Coating was performed for 1.5 h at 37 °C using 96-well MaxiSorp plates (Thermo Fisher Scientific) and fibrin-coated plates were dried at 37 °C overnight as described previously^17^.

### Recombinant SARS-CoV-2 spike protein binding on fibrin or fibrinogen

Fibrin- or fibrinogen-coated 96-well plates were washed with wash buffer (0.05% Tween-20 in PBS), and incubated with blocking buffer consisting of wash buffer with 5% bovine serum albumin (BSA) (Omnipure, Thermo Fisher Scientific) for 1 h at 25 °C. Serial dilutions of recombinant spike or S1(N501Y) were made in binding buffer (wash buffer containing 0.5% BSA). Recombinant spike or S1(N501Y) was added to the wells and incubated for 2 h at 37 °C. After washing five times with wash buffer, rabbit polyclonal anti-6× His tag antibody (ab137839, Abcam, 1:1,000) was added to the plates and incubated for 1 h at 25 °C. After washing, goat anti-rabbit IgG H&L (conjugated with horse radish peroxidase, HRP) (ab205718, Abcam, 1:1000) in wash buffer was added for 1 h at 25 °C. After the final wash, the HRP substrate 3,3′,5,5′-tetramethybenzidine (TMB; Sigma-Aldrich) was added into the wells. The reaction was quenched by adding 1 N hydrochloric acid, and the absorption was measured at 450 nm. Nonlinear regression curves were analysed using Graph Pad Prism 9 software to calculate *K*_d_ values using a one-site binding model.

### Fibrinogen peptide array and spike-binding epitope mapping

A custom PepStar Multiwell Fibrinogen Peptide Array comprising a synthetic peptide library with 390 15-mer peptides representing overlapping peptide scans (15/11) of the α, β and γ fibrinogen chains (UniProt: FIBA, P02671; FIBB, P02675; FIBG, P02679) was generated by JPT Peptide Technologies. The arrays were hybridized with recombinant-His-tagged trimeric spike (1 µg ml^−1^ in blocking buffer) for 1 h at 30 °C. The His-tag peptide (AGHHHHHH) was also immobilized on the peptide microarray as an assay control. Microarray slides were incubated for 1 h at 30 °C with Alexa 647 anti-6×His monoclonal antibody (MA1-135-A647, Invitrogen) diluted to 1 µg ml^−1^ in blocking buffer and dried. Before each step, microarrays were washed with washing buffer, 50 mM TBS-buffer including 0.1% Tween-20, pH 7.2. The assay buffer was LowCross buffer (Candor Bioscience). The slides were washed, dried and scanned with a high-resolution laser scanner at 635 nm to obtain fluorescence intensity profiles. The images were quantified to yield a mean pixel value for each peptide. To assess non-specific binding to the peptides and assay performance, a control incubation with secondary antibody was performed in parallel on each slide. The resulting images were analysed and quantified using spot-recognition software (GenePix, Molecular Devices). For each spot, the mean signal intensity was extracted (between 0 and 65,535 arbitrary units). Heat maps were computed and fluorescence intensities were colour-coded. Binding peptides were mapped onto the fibrinogen crystal structure (Protein Data Bank (PDB): 3GHG) using UCSF Chimera^58^. For the spike peptide array, 1, 0.1 or 0.01 µg ml^−1^ His-tagged recombinant human fibrinogen γ chain (Novus Bio) was hybridized with the SARS-CoV-2 spike Glycoprotein Variant Collection Peptide Microarray (JPT). Binding was detected using an anti-His secondary antibody conjugated to Alexa 647. Non-specific binding was detected using an anti-His secondary antibody only. Separately, 1, 0.1 or 0.01 µg ml^−1^ Alexa-647 fibrinogen (Invitrogen) was hybridized onto the spike Glycoprotein Variant Collection Microarray, and peptide binding was directly detected by fluorescence intensity in relative light units (RLU). A heat map was generated by using raw RLU for side-by-side comparison. Spike glycoprotein binding sites on fibrinogen were mapped using the PDB (6VSB).

### Peptide alanine scanning

Alanine scanning was performed with custom PepStar Multiwell microarrays (JPT) containing 60 peptides representing Ala substitutions of each residue on fibrinogen peptide γ_377–395_ (YSMKKTTMKIIPFNRLTIG). Human full-length IgG and His-tagged peptides were co-immobilized on the microarray slides as controls. His-tagged spike was applied at five concentrations (from 10 μg ml^−1^ to 0.001 μg ml^−1^) and incubated for 1 h at 30 °C. Two fluorescently labelled secondary antibodies specific to the His tag were added separately for 1 h. Washing and detection was performed as described above and data were analysed with respect to the original peptide. The signal after Ala substitution indicated whether a residue was involved in binding to spike.

### Structure preparation and homology modelling

The crystal structure of human fibrinogen (PDB: 3GHG) was fixed using the Structure Preparation application of the Compute module of MOE. The crystal structure of SARS-CoV-2 spike (PDB: 6VSB) has missing structural information for flexible loops. To correct these, the Homology Model application in the Protein menu of MOE 2022.02 software (Chemical Computing Group) was used, which includes: (1) initial partial geometry specification; (2) insertions and deletions; (3) loop selection and sidechain packing; and (4) final model selection and refinement. Homology models were inspected using MOE’s Protein Geometry stereochemical quality evaluation tools. The spike crystal structure (PDB: 6VSB) was prepared by assigning protonation and ionization states.

### Docking and calculation of energies of docked complexes

Docking of two proteins was performed by Dock application of Compute module of MOE, using the Protein-Protein function. The application generates a collection of docked configurations from the pool of possible binding positions using the rigid-body docking. To complete a docking procedure, the binding sites were identified based on the peptide array described above. Three potential binding sites were chosen for fibrinogen: (1) 119YLLKDLWQKRQ129 in the β-chain; and, in the γ-chain, (2) 163QSGLYFIKPLKANQQFLVY181 and (3) 364DNGIIWATWKTRWYSMKKTTMKIIPFNRLTIG395. For the ligand (spike protein) five sites were selected. NTD binding region: (1) 37YYPDKVFRSSVLHSTQDLFLPFFSNVTWFHAIHVSGTNGTKRFDNPVLPFNDGVYFASTEKSNIIRG103, (2) 229LPIGINITRFQTLLALHRSYLTP251 and (3) 305SFTVEKGIYQTSNF319; RBD region: (4) 341VFNATRFASVYAWNR355; and S2 domain: (5) 1049LMSFPQSAPHGVVFL1063. After receptor, ligand and docking sites were defined, parameters of Dock application of the Compute module of MOE were set to: refinement --Rigid Body, Poses --10. The application created 10 poses, analysed output scores, ligand docking energies and docked poses, and detected the best one; intermediate poses also are saved in a docking database file.

During the docking calculations the program presents 10 best energy complexes. After that, each of the complexes undergone the additional calculations of energy. A computational alanine scan of the fibrinogen molecules in each complex was also conducted with each of the residues in fibrinogen that were experimentally substituted to alanine were computationally substituted to alanine and modelled. The best model was selected on the basis of the lowest docking energy. The computational alanine scan generated the values of correlations between all values of energy for each amino acid substitution and experimental values of the parameter used for estimating the influence of each amino acid. The residues involved in the interaction of this computationally predicted complex were analysed using LigPlot+ v.2.2.

### i.v. injection of labelled spike S1(N501Y) and fibrinogen

Spike S1(N501Y) (AcroBiosystems) (20 μg) dissolved in 0.1 M PBS was fluorescently labelled using the Alexa Fluor 647 conjugation kit lightning link (Abcam). The Alexa-Fluor-647-labelled spike S1(N501Y) had a concentration of 1 mg ml^−1^. Retro-orbital injections of 0.1 ml of PBS solution containing 20 μg Alexa-647-conjugated spike S1(N501Y) and 30 μg Alexa-546-labelled human fibrinogen (Invitrogen) were performed under isoflurane anaesthesia (1 ml insulin syringe with a 30-gauge needle). The mice were perfused at 1 day after injection with heparinized PBS and fixed with 4% paraformaldehyde (PFA) and lungs were collected for clearing.

### 3DISCO clearing and light-sheet imaging

3DISCO lung tissue clearing was performed as described previously^59^. Mouse lungs were placed into a 20 ml scintillation glass vial and incubated in 20 ml of THF (Tetrahydrofuran, Roth, CP82.1) gradient in distilled water in a fume hood with gentle shaking at 50% once, 70% once, 80% once and 100% twice for 6 h for each step, followed by 3 h in dichloromethane (DCM, Sigma-Aldrich, 270997). The samples were immersed in BABB solution (benzyl alcohol + benzyl benzoate 1:2 (v/v), Sigma-Aldrich, 24122 and W213802) until optical transparency. Lung tissues were imaged using Imspector Pro v.7.0.98 and the LaVision BioTec Ultramicroscope II light-sheet microscope in a quartz cuvette filled with ethyl cinnamate (ECi) (Sigma-Aldrich). For imaging, MVX10 zoom body (Olympus) with a ×2 objective (pixel size, 3.25 µm for *x* and *y*) at magnification from ×0.63 up to ×1.6 was used. Up to 1,400 images were taken for each lung using a *z*-step size of 3.5 µm, and light-sheet numerical aperture of 0.111 NA. Band-pass emission filters (mean nm/spread) were used, depending on the excited fluorophores: 525/50 for autofluorescence; 595/40 for AF546; and 680/30 for AF647. The exposure time was 10 ms for single channel and 25 ms for multichannel acquisition. Imaris v.9.5.0 (Bitplane) was used for 3D rendering. Pixel dimensions were updated from the non-reduced 16-bit image metadata. Surface objects in Imaris was used to 3D render focal depositions and spike distribution in representative volumetric ROIs.

### Plasmin digestion of fibrin

Before clotting, 3 μM fibrinogen was incubated with 9 μM recombinant spike protein at 37 °C for 1 h in 20 mM HEPES, pH 7.4, 137 mM NaCl, 5 mM CaCl_2_. Thrombin was added at a final concentration of 1.5 U ml^−1^. Fibrin clots were allowed to form in Eppendorf tubes for 2 h at 37 °C. Then, 5 μl of 100 μg ml^−1^ plasmin (Millipore) was added to each tube on top of the clot. All of the samples were incubated at 37 °C for 0, 1, 2, 4 and 6 h; digestion was quenched by adding sodium dodecyl sulfate–polyacrylamide gel electrophoresis (SDS–PAGE) loading buffer with reducing agent. The samples were heated at 85 °C for 20 min, and aliquots (equivalent to 100 ng fibrinogen) were separated by SDS–PAGE on 4–12% Bis-Tris gels, transferred to PVDF membranes and analysed for anti-human fibrinogen (F4200-06, US Biological, 1:2,000) by western blotting. The band intensities of each protein species (that is, γ–γ dimer, β-chain) were analysed using ImageJ and normalized to the corresponding bands at the 0 h timepoint. The loading control for the western blot is the timepoint 0 before the addition of plasmin to the fibrin clot.

### Competitive ELISA of 5B8 versus the spike for binding to fibrin

A 5B8-huFc antibody was synthesized by Fc swap of the mouse IgG2b Fc of 5B8^18^ with human IgG1 Fc. 96-well ELISA plates (Greiner) were coated with 25 μg ml^−1^ IgG-depleted fibrin and incubated in blocking buffer as indicated for binding assays for 1 h before addition of 50 μl per well of 5B8-huFc antibody. Human plasminogen-free fibrinogen was depleted from IgG as described previously^17^. The antibody was diluted at threefold concentrations from 0.0002 μM to 15 μM in PBS with 0.5% BSA and 0.05% Tween-20 (diluent). For the competition ELISA without preincubation, 5B8-huFc was incubated together with 150 nM trimeric spike in diluent (100 μl total volume) for 2 h at 37 °C on fibrin plates. For the ELISA with antibody preincubation, 50 μl of 5B8-huFc was incubated on fibrin plates for 2 h at 37 °C, followed by addition of 50 μl of 150 nM trimeric spike to the antibody and incubation for 2 h at 37 °C. This was followed by incubation with HRP-coupled anti-His tag antibody (MAB050H, R&D Systems, 1:2,000) for 1 h at 25 °C. The ELISA was developed by incubation with TMB/E substrate (Chemicon-Millipore), and the absorbance was measured at 450 nm using the Synergy H4 plate reader (BioTek).

### ROS detection

BMDM culture and ROS detection using 5 µM DHE (Invitrogen) were performed as described previously^17,60^. In brief, cells were plated on 96-well black μ-clear-bottomed microtitre plates (Greiner Bio-One) precoated with 12.5 µg ml^−1^ fibrin with or without recombinant spike (0.168, 1.68 and 3.36 µM), spike PVs or bald PVs. For fibrin inhibition, 5B8 or IgG2b (each 20 μg ml^−1^) (MPC-11, BioXCell) was added in fibrin with or without 3.36 µM recombinant spike-coated wells 2 h before plating. Cells were incubated on fibrin for 24 h and DHE fluorescence was detected at 518 nm/605 nm using the SpectraMax M5 microplate reader. As macrophage activation can be influenced by cell culture conditions, heat-inactivated fetal bovine serum and macrophage colony-stimulating factor were batch tested as described previously^60^. As the activity of PVs can be influenced by freeze–thaw cycles, all of the experiments were performed with virions that had been freshly thawed and kept at 37 °C. Refrozen virion samples were not used.

### Immunoprecipitation

To test interaction of fibrinogen with His-tagged spike, the Pierce co-immunoprecipitation kit (Thermo Fisher Scientific) protocol was used with original immunoprecipitation/lysis buffer and modifications. Spike and fibrinogen were mixed at a molar ratio of 2:1 in 800 μl of immunoprecipitation buffer (50 mM Tris, pH 8.0, 5% glycerol, 1% NP-40, 100 mM NaCl) supplemented with 100 × EDTA-free Halt protease inhibitor (Thermo Fisher Scientific) and then rotated at 37 °C for 1 h. Resin beads conjugated with the anti-fibrinogen antibody (SAFG-AP, Enzyme Research Laboratories, 1:1,000) were added to the mixture and rotated at 37 °C for another 2 h. The bound proteins were eluted in 60 µl of EB solution and neutralized with 1/10 volume of 1 M Tris, pH 9.0. The wash buffer and EB solution were warmed to 37 °C in advance. The eluted proteins were separated by SDS–PAGE on 4–12% gels, transferred to PVDF membranes (Invitrogen) and incubated with rabbit anti-spike antibody (632604, GeneTex, 1:1,000) and sheep anti-fibrinogen antibody (SAFG-AP, Enzyme Research Laboratories, 1:1,000) and then with HRP-conjugated anti-rabbit (111-035-144, Jackson ImmunoResearch; 1:10000) and anti-sheep (HAF016, R&D Systems; 1:5,000) secondary antibodies. For immunoprecipitation of spike PVs, spike antibodies (GTX635693, GeneTex; 1:1,600) recognizing SARS-CoV-2 spike (S2) were used. For spike PV immunoblot, anti-spike (632604, GeneTex, 1:1,000) and anti-p24 Gag (detecting p55, 1:100) antibodies donated to the Greene laboratory by Beckman Coulter^61^ and anti-Vpr (8D1, Cosmo Bio, 1:200) antibodies were used. Protein bands were detected using Immobilon Forte Western HRP substrate (Sigma-Aldrich) and the ChemiDoc imaging system (Bio-Rad).

### SARS-CoV-2 culture and in vivo infection

To assess SARS-CoV-2 infection in vivo, viral stocks of SARS-CoV-2 B.1.351 (Beta) and SARS CoV-2 B.1.617.2 (Delta) were prepared on Vero cells expressing transmembrane protease serine 2 (TMPRSS2) and ACE2 (Vero-TMPRSS2-ACE2)^47^ provided by A. Creanga and B. Graham at NIH and stored at −80 °C until used. Experiments involving Beta were performed on female and male WT C57BL/6, *Fga*^–/–^ and *Fgg*^γ390–396A^ mice (6–7 months of age). The Beta strain contains the K417Y, E484K and N501Y substitutions in the spike RBD and binds to mouse ACE2 inducing active infection in a range of experimental mouse strains^62–64^. Experiments using Delta were performed on female and male 4–5 month old K18-hACE2 mice. For the infection, the animals were anaesthetized using 100 mg per kg ketamine mixed with 10 mg per kg xylazine through intraperitoneal injection. Anaesthetized mice received i.n. administration of an infectious inoculum of virus in 50 μl of serum-free DMEM. For each experiment, lung and brain tissues were collected. Left lung lobes and one brain hemisphere from each animal were placed in 4% PFA for fixation and histological processing. The remaining lung tissue was roughly chopped and processed for homogenates in prefilled zirconium bead tubes (Benchmark Scientific). Homogenates were stored at −80 °C. The remaining brain hemispheres were flash-frozen and stored at −80 °C. All aspects of this study were approved by the office of Environmental Health and Safety at UCSF before initiation. Work with SARS-CoV-2 was performed in a biosafety level 3 laboratory by personnel equipped with powered air-purifying respirators.

### Plaque assay

Lung homogenates were assessed for viral concentration by plaque assay. In brief, Vero-TMPRESS2-ACE2 cells were plated onto 12-well plates at a concentration of 2 × 10^5^ cells per well. Homogenates were added to the cells in a dilution series of 10^1^, 10^2^, 10^3^, 10^4^, 10^5^ and 10^6^ in serum-free DMEM. The homogenate dilutions were incubated on the cells for 1 h, and the media in the wells was then overlaid with 2.5% Avicel (Dupont, RC-591). Cells were incubated for 72 h, then the overlay was removed and the cells were fixed in 10% formalin for 1 h, and stained with crystal violet to visualize PFU.

### Production of spike PVs

HEK293T cells (3.75 × 10^6^) were plated in a T175 flask and transfected 24 h later with 90 μg of polyethyleneimine (PEI; Sigma-Aldrich), 30 μg of HIV-1 NL4-3 ∆ Env eGFP (NIH AIDS Reagent Program) or 3.5 μg of pCAGGS SARS-CoV-2 trimeric spike glycoprotein (NR52310, BEI Resources) in a total of 10 ml of Opti-MEM medium (Invitrogen). The next day, the medium was replaced with DMEM10 complete medium, and the cells were incubated at 37 °C in 5% CO_2_ for 48 h. The supernatant was then collected, filtered with 0.22 µm Steriflip filters (EMD, Millipore) and ultracentrifuged at 25,000 rpm for 1.5 h at 4 °C. The concentrated supernatant was removed, the pellets (viral particles) were resuspended in cold 1× PBS containing 1% fetal bovine serum and aliquots were stored at −80 °C in a biosafety level 3 laboratory. For the production of control viral particles not expressing the spike glycoprotein (bald), the same procedure was used but with the omission of the pCAGGS SARS-CoV-2 spike vector transfection. HIV Env pseudotyped viral particles were also produced with the same procedure, using an HIV89.6 Env dual tropic (X4 and R5) expression vector (NIH AIDS Reagent Program) instead of the spike expression vector.

### In vivo administration of SARS-CoV-2 spike PVs

Mice were anaesthetized with isoflurane and spike PVs or bald PVs (control) (100 µl) were slowly injected into the retro-orbital plexus with a BD 0.3 ml insulin syringe attached to a 29-gauge needle. After 3 min, the needle was slowly withdrawn, and the mice were allowed to recover. As the activity of PVs can be influenced by freeze–thaw cycles, all of the experiments were performed with virions that had been freshly thawed and kept at 37 °C. Refrozen virion samples were not used. SARS-CoV-2 spike PVs were administered to 3- to 4-month-old mice.

### 5B8 penetration in the CNS and target engagement after SARS-CoV-2 infection

C57BL/6 mice (4–5 months of age) were infected with 10^4^ PFU of SARS-CoV-2 B.1.351 (Beta). On 5 and 7 d.p.i, mice were given intraperitoneally 30 mg per kg of the 5B8-huFc antibody. On 7 d.p.i, mice were perfused with saline followed by fixation with 4% PFA. Subsequently, the brains were post-fixed in the same fixative and cryoprotected in 30% sucrose. The brain hemispheres were frozen in OCT and sectioned (10 µm sections). Sagittal brain sections were incubated with 0.1% Sudan Black (dissolved in 70% ethanol) for 10 min, permeabilized/blocked with 3% BSA and 3% NDS in PBS containing 0.1% Triton X-100 for 1 h. The sections were incubated overnight with an antibody to fibrinogen (1:2,000), followed by Alexa Fluor 594 donkey anti-rabbit IgG (1:1,000; Jackson ImmunoResearch) for 1 h. To detect 5B8-huFc antibody in the brain, the sections were stained with F(ab′)2-donkey anti-human IgG (H+L) cross-adsorbed secondary antibody, FITC (ab102424, Abcam, 1:300) for 1 h. The sections were covered with glass coverslips, sealed with ProLong Diamond Antifade Mounting reagent (Thermo Fisher Scientific) and kept at 4 °C until imaging.

### Fibrin 5B8 antibody treatment

For prophylactic pharmacological treatment of SARS-CoV-2 B.1.351 (Beta) infection, anti-fibrin antibody 5B8^17^ or an isotype-matched IgG2b (MPC-11, BioXCell) control were administered intravenously by retro-orbital injection at 30 mg per kg in 5- to 6-month-old C57BL/6 mice. Then, 1 h later, the mice were given 10^4^ PFU of Beta through the i.n. route in a final volume of 50 μl. Beta-infected mice were euthanized at 3 days for histological analysis. For SARS CoV-2 B.1.617.2 (Delta) infection, 4- to 5-month-old K18-hACE2 mice were given 5B8 or IgG2b intravenously through retro-orbital injection at 30 mg per kg 1 h before Delta infection and every 48 h intraperitoneally, and were euthanized at 3 d.p.i. For therapeutic treatments, 5B8 or IgG2b were given intraperitoneally at a dose of 30 mg per kg at 1 d.p.i. with 10^3^ PFU of Beta in 5- to 6-month-old C57BL/6 mice or Delta in 4- to 5-month-old K18-hACE2 mice as described above, and every 48 h thereafter, intraperitoneally. The animals were euthanized at 7 or 9 d.p.i. For spike PVs, 5B8 or IgG2b isotype control were given intravenously to C57BL/6 mice by retro-orbital injection at 30 mg per kg 15 min before injection of PVs. Generation of 5B8 and dose of administration have been described previously^17^. Administration of mouse monoclonal antibodies intraperitoneally provides sustained release of antibody into the bloodstream and thus is commonly used to assess preclinical efficacy for antibodies that will eventually be delivered intravenously in the clinic^65–67^.

### Histology and immunohistochemistry

Histopathological analysis in mouse lung and brain was performed on frozen or paraffin sections^17,68,69^. Serial sections were not collected in the study. Lung sections were stained with haematoxylin and eosin and trichrome. The following antibodies were used: rabbit anti-SARS-CoV-2 nucleocapsid (GTX135357, GeneTex, 1:500), mouse anti-SARS-CoV-2 spike (1A9, GeneTex, 1:100), sheep anti-fibrinogen (F4200-06, US Biological, 1:300), rabbit polyclonal anti-fibrinogen (gift from J. Degen, 1:500), rat anti-mouse/human Mac2 (M3/38, Cedarlane, 1:500), mouse anti- gp91^phox^ (53/gp91-phox, BD Biosciences, 1:500), rat anti-mouse CD335 (NKp46) (29A1.4, BD Biosciences, 1:500), mouse anti-NK1.1 (PK136, Invitrogen, 1:250) and rabbit anti-granzyme A (PA5-119160, Invitrogen, 1:500). Brains were cut with a cryostat into 30-μm-thick frozen sections for free-floating immunostaining. The following antibodies were used: rabbit anti-IBA1 (019-19741, Wako, 1:1,000), rat anti-mouse CD68 (FA-11, BioLegend, 1:500), guinea pig anti-NeuN (A60, Sigma-Aldrich, 1:500), rat anti-myelin basic protein (ab7349, Abcam, 1:100) and rabbit anti-calbindin (CB38a, Swant; 1:5,000). The tissue sections were washed in PBS and incubated in a blocking and permeabilization buffer consisting of PBS supplemented with 0.2% Triton X-100 and 5% BSA for 1 h at 25 °C. For mouse primary antibodies, the sections were incubated in M.O.M. (Mouse on Mouse Immunodetection Kits, Vector Laboratories) mouse IgG blocking reagent diluted in PBS containing 0.2% Triton X-100 and 5% BSA, and then with M.O.M. diluent for 5 min at room temperature. The sections were rinsed twice with PBS containing 0.1% Triton X-100 and incubated overnight with primary antibodies at 4 °C. All of the tissue sections were washed with PBS containing 0.1% Triton X-100 and incubated with the following secondary antibodies: goat anti-rabbit Alexa Fluor 488 (A-11008, Thermo Fisher Scientific, 1:1,000), goat anti-mouse Alexa Fluor 568 (A-110041, Thermo Fisher Scientific, 1:1,000) or goat anti-rat Alexa Fluor 647 (A-21247, Thermo Fisher Scientific, 1:1,000), and stained with DAPI. The sections were mounted on frosted microscopy slides (Thermo Fisher Scientific), covered with glass coverslips, sealed with ProLong Diamond Antifade Mounting reagent (Thermo Fisher Scientific) and kept at 4 °C until imaging.

### Confocal microscopy

Tissue sections were imaged using a laser-scanning confocal microscope FLUOVIEW FV3000RS “Snow Leopard” (Olympus) or Fluoview FV1000 (Olympus), a 40 × and 0.8 NA water-immersion lens or 60× oil-immersion UPLSAPO objective (NA = 1.35) and FV31S-SW software v.2.3.2.169 (Olympus). Individual channels were captured sequentially with a 405 nm laser and a 430/70 spectral detector for DAPI, a 488 nm laser and a 500/40 spectral detector for Alexa Fluor 488, a 561 nm laser and a 570/620 high-sensitivity detector for Alexa Fluor 568, and a 650-nm laser and a 650/750 high-sensitivity detector (Olympus TruSpectral detector technology) for Alexa Fluor 647. Captured images were processed with Fiji v.2.1.0/ImageJ v.1.53c.

### Image analysis

To analyse microglia after stereotaxic injections of fibrinogen, spike or PVs, the corpus callosum within five rostrocaudally spaced coronal brain sections was selected for quantification^17^. To quantify IBA1, CD68, calbindin or NeuN^+^ cells in mice infected with Beta or Delta, three areas in the hippocampus (for IBA1 or CD68) or two areas in the cortex (for calbindin or NeuN) were selected on three mediolaterally spaced sagittal brain sections, ensuring consistency in anatomical regions per mouse. For lung pathology in Beta-infected mice, six or seven representative areas were chosen from three lung sections. N protein-positive areas were selected for collagen quantification. Lung pathology in mice injected with PVs was performed on five representative areas selected from three lung sections. Immunostained cells were counted with Jupyter Notebook in Python 3. In brief, an arbitrary threshold was manually set and used for all images in the dataset. The total number of cells per image was estimated using the function peak_local_max from the open-source skimage Python image-processing library, which returns the coordinates and number of local peaks in an image (https://scikit-image.org/docs/dev/api/skimage.feature.html#skimage.feature.peak_local_max). Fibrinogen immunoreactivity was quantified using Fiji (ImageJ) as described previously^70^. Python image processing was used to colocalize fibrinogen and spike protein in lung tissues. In brief, a Jupyter Notebook was written to estimate the amount of fluorescence signal overlap between spike and fibrinogen in lung tissues. The Ostu filter from the skimage Python image-processing library was used to threshold each image labelled with spike and fibrinogen (https://scikit-image.org/docs/0.13.x/api/skimage.filters.html#skimage.filters.threshold_otsu). After thresholding, each set of images was compared, and pixels were compartmentalized into 4 categories: spike and fibrinogen overlap, spike signal only, fibrinogen signal only and no signal. In each image, the total number of pixels in an image and the number of pixels with signal for spike only, fibrinogen only or both were computed. Correlations were calculated using FOVs from all images collected as indicated in Extended Data Figs. 1b,c and 9f to assess the distribution across the dataset. All images selected for the figures are representative of the quantification of immunostaining for each experimental group.

### Bulk RNA-seq

Lungs (3 d.p.i.) were isolated and snap-frozen with liquid nitrogen and stored at −80 °C. RNA samples were isolated using the RNeasy Plus Mini Kit (Qiagen). Generation of cDNA, sequencing, quality control of raw count, mapping and counting was performed as described^21,60^. The samples used for gene expression analysis were confirmed for viral load by quantitative PCR in lung tissue for expression of N5 specific for Beta variant. Samples with poor RNA quality or no viral load were excluded from further analysis. All of the samples that passed RNA quality control were included in the study. A minimum of three replicates per group was used, and genes with less than 0.1 counts per million (CPM) were filtered out from the study. Normalization was then performed using calcNormFactors, and differentially expressed genes were determined using edgeR^71^. The false-discovery rate (FDR) was calculated using the Benjamini–Hochberg method. For NK cell RNA-seq, adjusted *P* < 0.1 (two-sided quasi-likelihood *F*-test with Benjamini–Hochberg correction) was used for visualization in Fig. 3a. The CPM of each gene was normalized across all of the samples to generate *z*-scores for heat maps of gene expression. Differentially expressed genes significantly changed in uninfected mice were not included in the analysis. For pathway analysis, gene lists were ranked using log_2_-transformed fold change of differentially expressed gene between two groups. Fibrin-induced macrophage scRNA-seq data were obtained from ref. ^21^ (GSE229376). GSEA was performed using GSEA v.4.2.3 with 1,000 times permutation and collapsing mouse genes to the chip platform Mouse_Gene_Symbol_Remapping_Human_Orthologs_MSigDB.v7.5.1.chip. The MSigDB gene sets: H: Hallmark and C2: CP: Canonical pathways (KEGG, REACTOME, WikiPathways) were used for pathway analysis. The fibrin NK suppression network was generated using Cytoscape (v.3.7.2)^72^. Using differentially altered pathways generated by GSEA (described earlier), the network was visualized using the default setting of EnrichmentMap.

### NK cell depletion and characterization

NK cells were purified from splenocytes of C57BL/6 mice using the NK cell isolation kit (Miltenyi Biotec). NK cells were stimulated with IL-15 (50 ng ml^−1^, BioLegend) for 4 days with or without fibrin. Flow cytometry staining and analyses were performed as described previously^21,60^. For NK cell surface and intracellular staining, NK cell suspensions were first incubated with TruStain FcX PLUS (S17011E, BioLegend) for 15 min at 4 °C, then stained with surface markers for 30 min at 4 °C. Cells were then fixed and permeabilized using the BD Fixation/Permeabilization Kit (554714, BD). Intracellular markers were incubated for 1 h at 4 °C and analysed using the LSR Fortessa flow cytometer (BD Biosciences) the same day. For IFNγ staining, NK cells were incubated with phorbol 12-myristate 13-acetate (P8139, Sigma-Aldrich) and ionomycin (I0634, Sigma-Aldrich) for 4 h in the presence of brefeldin A (B7651, Sigma-Aldrich) followed by surface staining and fixation/permeabilization protocol described above. Anti-IFNγ antibodies were incubated in perm/wash buffer overnight, and then analysed with LSR Fortessa flow cytometer (BD Biosciences) the same day. Antibodies were as follows: NK1.1-FITC (S17016D, BioLegend, 1:200), IFNγ-PE (XMG1.2, BioLegend, 1:200), granzyme B-PerCP/Cy5.5 (QA16A02, BioLegend, 1:200), Ki-67-PE (16A8, BioLegend,1:200), CD45-Brilliant Violet BUV737(30-F11, BD, 1:200), CD11b-Brilliant Ultraviolet 395 (M1/70, BD, 1:200), CD335-Brilliant Violet 421 (clone 29A1.4, BioLegend,1:100), CD54-PE (YN1/1.7.4, BioLegend, 1:200), CD314-APC (CX5, BioLegend, 1:200), LIVE/DEAD Fixable Aqua Dead Cell Stain Kit (L34957, Thermo Fisher Scientific, 1:500). All data were processed using FlowJo v.10.7.1 (BD Biosciences). Doublets and dead cells were excluded before analysis of NK cell phenotypes. NK cells were gated as CD45^+^CD3^−^NK1.1^+^. For NK cell depletion, anti-mouse NK1.1 (PK136, BioXCell), which depletes NK cells^73–75^, or isotype control IgG2a (C1.18.4, BioXcell) were administered intraperitoneally at 8 mg per kg at 3 and 1 days before infection of 5- to 7-month-old mice.

For bulk RNA-seq analysis of mouse NK cells, purified NK cells from splenocytes of C57BL/6 mice were stimulated with IL-15 (50 ng ml^−1^, BioLegend) for 4 days with or without fibrin. NK cells were stained with anti-CD3 (145-2C11, BD, 1:200), anti-NK1.1 (S17016D, BioLegend, 1:200), anti-CD45 (30-F11, BioLegend, 1:200) and aqua live/dead fixable dye on ice for 20 min. The CD45^+^CD3^−^NK1.1^+^ live NK cells were sorted into 1.5 ml tubes with 1 ml of Buffer RLT Plus with 1% β-mercaptoethanol. RNA samples were prepared using the RNeasy Plus Micro Kit according to the manufacturer’s instructions. The cDNA library generation, quality control, sequencing and downstream analysis are performed as above.

### Sample preparation for MS analysis

Human NK cells were isolated from freshly collected PBMCs (AllCells) using the NK cell Isolation Kit, Human (Miltenyi Biotec). In total, 5 × 10^6^ NK cells were plated on each well of a six-well plate treated with or without fibrin for 1 h at 37 °C. Phosphoproteomic analysis was performed as described previously^21,32^. The samples were washed twice with cold PBS, lysed in 6 M guanidine hydrochloride (Sigma-Aldrich), then boiled at 95 °C for 5 min, and stored on ice until sonication. Lysed samples were sonicated using a probe sonicator for 15 s at 10% amplitude, and protein was quantified by Bradford assay. Approximately 500 µg of protein sample was used for further processing, starting with reduction and alkylation using a 1:10 sample volume of tris-(2-carboxyethyl) (TCEP) (10 mM final) and 2-chloroacetamide (40 mM final) for 5 min at 45 °C with shaking at 1,500 rpm. Before protein digestion, the 6 M guanidine hydrochloride was diluted sixfold with 100 mM Tris-HCL (pH 8) to permit trypsin activity. Trypsin was then added at a 1:100 (w/w) enzyme:substrate ratio and placed in a thermomixer at 37 °C overnight (16 h) with shaking at 800 rpm. After digestion, 10% trifluoroacetic acid (TFA) was added to each sample to reach a final pH of 2. The samples were desalted using a vacuum manifold with 50 mg Sep Pak C18 cartridges (Waters). Each cartridge was activated with 1 ml 80% acetonitrile/0.1% TFA, then equilibrated with 3 × 1 ml of 0.1% TFA. After sample loading, the cartridges were washed with 3 × 1 ml of 0.1% TFA, and the samples were eluted with 1 × 0.8 ml 50% acetonitrile/0.25% formic acid. The samples were dried by vacuum centrifugation. The High-Select Fe-NTA phosphopeptide enrichment kit (Thermo Fisher Scientific) was used according to the manufacturer’s instructions with minor modifications for phosphopeptide enrichment. In brief, the samples were suspended in approximately one-third of the recommended binding/wash buffer volume (70 µl). After equilibrating the spin column, the resin slurry was resuspended in 210 µl of binding/wash buffer and divided into thirds. Each third of the resin was used for one sample. Tryptic peptides were mixed with the resin in a separate protein LoBind tube (Eppendorf) and incubated for 30 min (at room temperature) on a thermomixer at 800 rpm. The samples were transferred on top of a 20 µl filtered tip, washed three times with binding/wash buffer and once with HPLC-grade water. The bound phosphopeptides were eluted with 70 µl elution buffer, and the pH was brought down immediately to nearly three with formic acid (10% (v/v) in HPLC-grade water). All of the samples were dried by vacuum centrifugation and stored at −80 °C until further analysis.

### MS proteomics data acquisition

Dried phosphopeptides were resuspended in 0.1% (v/v) formic acid (Sigma Aldrich) in water (HPLC grade, Thermo Fisher Scientific) and analysed on the timsTOF HT mass spectrometer (Bruker Daltonics), paired with a Vanquish Neo ultra-high-pressure liquid chromatography system (Thermo Fisher Scientific). The samples were directly injected onto a PepSep C18 reverse-phase column (15 cm, 150 µm inner diameter, 100 Å pore size, 1.5 µm particle size with UHP inlet, Bruker Daltonics) connected to a captive spray emitter (ZDV, 20 µm, Bruker Daltonics). Mobile phase A consisted of 0.1% (v/v) formic acid in water (HPLC grade, Thermo Fisher Scientific) and mobile phase B consisted of 0.1% (v/v) formic acid in 100% acetonitrile (HPLC grade, Thermo Fisher Scientific). Peptides were separated on a gradient from 3% to 25% mobile phase B over 47 min, followed by an increase to 45% B over 8 min, then to 95% over 1 min, and held at 95% B for 4 min for column washing at a flow rate of 200 nl min^−1^. Eluted peptides were ionized in a CaptiveSpray source (Bruker Daltonics) at 1,700 V. Raw data were acquired in data-independent acquisition coupled with parallel accumulation–serial fragmentation (dia-PASEF) mode with an optimized isolation window scheme in the *m*/*z* versus ion-mobility plane for phosphopeptides. The ion accumulation time and ramp times in the dual TIMS analyser were set to 100 ms each. For dia-PASEF, in the ion mobility (1/K0) range 0.6 to 1.50 Vs cm^−2^, the collision energy was linearly decreased from 59 eV at 1/K0 = 1.6 Vs cm^−2^ to 20 eV at 1/K0 = 0.6 Vs cm^−2^ to collect the MS/MS spectra in the mass range 400.2 to 1,399.3 Da. The estimated mean cycle time for the dia-PASEF windows was 1.38 s. The raw files were processed with Spectronaut (v.18.5, Biognosys) using its library-free DIA analysis with directDIA+ (Deep) search algorithm. Carbamidomethylation (cysteine) was set as a fixed modification for database search. Acetylation (protein N-term), oxidation (methionine), and phosphorylation (serine, threonine, tyrosine) were set as variable modifications. Reviewed human protein sequences (downloaded from UniProt, 6 October 2023) were used for spectral matching. The FDRs for the PSM, peptide and protein groups were set to 0.01, and the minimum localization threshold for PTM was set to zero. For MS2 level area-based quantification, the cross-run normalization option was unchecked (normalization was performed later using MSstats, see below), and the probability cut-off was set to zero for the PTM localization. We detected between 4,000 and 7,000 phosphorylated peptides per sample with an average percentage of phosphorylated to non-phosphorylated peptides of 73%.

### Computational analysis of phosphoproteomics

Quantification of phosphorylation differences was performed using artMS as a wrapper around MSstats^76^, through functions artMS::doSiteConversion and artMS::artmsQuantification with the default settings. All peptides containing the same set of phosphorylated sites were grouped and quantified together into phosphorylation site groups. One sample outlier in intensity and peptide detection was discarded before quantitative analysis; unstimulated (mock) 1 h (PRIDE sample ID TOF01641_2_1_1683). For both phosphopeptide and protein abundance MSstats pipelines, MSstats performs normalization by median equalization, no imputation of missing values and median smoothing to combine intensities for multiple peptide ions or fragments into a single intensity for their protein or phosphorylation site group. Lastly, statistical tests of differences in intensity between infected and control timepoints were performed. When not explicitly indicated, we used the default settings for MSstats for adjusted *P* values. By default, MSstats uses the Student’s *t*-tests for *P* value calculation and the Benjamini–Hochberg method of FDR estimation to adjust *P* values. Kinase activities were estimated using known kinase–substrate relationships from the OmniPath database^77^. Kinase activities were inferred as a *z*-score calculated using the mean log_2_-transformed fold change of phosphorylated substrates for each kinase in terms of standard error (*Z* = [*M* − *μ*]/s.e.), comparing fold changes in phosphosite measurements of the known substrates against the overall distribution of fold changes across the sample. To compare all phosphorylation sites across experimental groups as previously described^32^, a *P* value was also calculated from log_2_-transformed fold changes of all detected phosphorylation sites using a two-tailed *Z*-test method as shown in Fig. 3c, Extended Data Fig. 7b and Supplementary Tables 8–10. Network reconstruction and enrichment analysis of phosphoproteomics data were performed as described previously^22^.

### Nanostring analysis

Formalin-fixed paraffin-embedded (FFPE) tissue was scrapped off into a 1.5 ml Eppendorf tube and deparaffinized with 1 ml of xylene for 2 min and then pelleted and washed with 1 ml of 100% ethanol. The samples were pelleted and incubated at room temperature until all of the residual ethanol had evaporated. Tissues were digested and RNA samples were isolated using the RNeasy FFPE Kit (Qiagen). The quantity was determined using the Nanodrop (Thermo Fisher Scientific) and the quality of RNA was determined on the Agilent Bioanalyzer. All of the samples passed quality control (>50% of RNA larger than 250 nucleotides). Gene expression assays were performed on the Nanostring nCounter machine with NS_Mm_HostResponse_v1.0 codeset. The raw data were processed and normalized counts, unadjusted *P* values and log_2_-transformed fold change values were generated with nSolver using two-tailed unpaired *t*-tests. For pathway analysis, the normalized counts of each gene were normalized across all of the samples to generate a *z*-score for heat maps of gene expression. The average *z*-score for each genotype was used for the heat map. Significantly downregulated genes between the 5B8 and IgG2b treated group (*P* < 0.05) were on clusterProfiler to determine significantly downregulated pathways using the enrichGO function. The top 20 significantly downregulated pathways were used to generate the network.

### Stereotactic injection of fibrinogen and spike

Fibrinogen was stereotactically injected into the brain as described previously^35^. Mice were anaesthetized with isoflurane and placed into a stereotaxic apparatus (Kopf Instruments). Alexa Fluor 488 human fibrinogen (Thermo Fisher Scientific) was dissolved in 0.1 M sodium bicarbonate (pH 8.3) at 25 °C to 1.5 mg ml^−1^ (ref. ^78^), mixed with spike (4.6 mg ml^−1^), spike PVs (0.1 mg ml^−1^), bald PVs (0.1 mg ml^−1^) or PBS control (1:1 ratio), and incubated at 37 °C for 15 min; 1.5 μl of the mixture was stereotactically injected at 0.3 μl min^−1^ with a 10 μl Hamilton syringe and a 33 gauge needle into the corpus callosum of 4- to 5-month-old C57BL/6 mice^35^. Mice were anaesthetized with avertin and transcardially perfused with 4% PFA in PBS. The brains were removed, post-fixed in 4% PFA overnight at 4 °C, processed with 30% sucrose, cut into 30 μm coronal sections and processed for immunohistochemistry. Images were acquired on the Axioplan II epifluorescence microscope (Zeiss) with Plan-Neofluar objectives (×10/0.3 NA). Images of similar anatomical locations were quantified using NIH ImageJ (v.1.50).

### RNA in situ hybridization with immunohistochemistry

RNA in situ hybridization with immunohistochemistry was performed on brain sections from mice infected with Delta using RNAscope Multiplex Fluorescent Assay (ACD Bio) according to the manufacturer’s protocol for FFPE tissue. In brief, tissue was deparaffinized and incubated in 3% hydrogen peroxide for 10 min, then subjected to antigen retrieval by boiling in RNAScope Target Retrieval Solution (ACD Bio) for 1 h. The samples were permeabilized with RNAScope Protease Plus reagent (ACD Bio) for 30 min at 40 °C. RNA probes were hybridized to tissue for 2 h at 40 °C. Oligonucleotide probes for mouse *Trem2*, *Cst7* and *Spp1* were designed by ACD Bio (498711-C3, and 435191-C3, respectively). Probe signals were amplified using the RNAScope Multiplex Fluorescent Reagent Kit v2 (ACD Bio) and detected with TSA Vivid Fluorophore 570 (Tocris, 7526). Tissue sections were stained for one RNA probe and counterstained for IBA1 (234 308, Synaptic Systems, 1:500) using the RNA-Protein Co-Detection Ancillary Kit (ACD Bio). The slides were imaged using the Zeiss Axioplan 2 epifluorescent microscope at ×20 and images were analysed using ImageJ (NIH). IBA1-postive microglia in each image were manually counted. Dense clusters of *Trem2*, *Cst7* or *Spp1* mRNA overlapping with IBA1 signal indicate microglia expressing disease-associated genes.

### Statistical analysis

All values are reported as mean ± s.e.m. The Shapiro–Wilk normality test^79^ was used to evaluate the normal distribution of the data. The equality of variance assumption was verified for both the responses in the natural and logarithmic scales using the Brown–Forsythe test^80^. Comparisons between two matched-paired groups, where the assumption of normal distribution for the differences of paired responses was met, were performed using paired *t*-tests. *P* values for comparisons between two independent groups were calculated using Mann–Whitney *U*-tests in the case of non-normally distributed data for which the equal variance assumption was not violated. For comparisons involving more than two groups, one- or two-way ANOVA followed by Tukey’s post hoc test for multiple comparisons was used for data meeting normal distribution and equal variance assumptions. When the assumption of equal variance was violated, Welch’s *t*-tests were applied to log_10_-transformed response values, and the resulting raw *P* values were corrected for multiple testing using the Holm method^57^. For the survival analysis and weight change data, *P* values were calculated using the log-rank (Mantel–Cox) test and mixed-effects model, respectively. Sample sizes were determined by previous studies rather than statistical approaches. For all in vivo experiments, mice were randomized and experiments were conducted in a blinded manner to the mouse genotype, antibody or PV administration. Genotype and treatment assignment were revealed after image quantification. For bulk RNA-seq and Nanostring experiments, both mouse genotype and antibody treatment were blinded. SEM imaging and image acquisition were performed blinded to test conditions. Biochemical studies of the binding of fibrinogen to spike were performed in the Akassoglou laboratory and independently validated in the Greene laboratory and Assay Development and Drug Discovery Core with similar results.

### Reporting summary

Further information on research design is available in the Nature Portfolio Reporting Summary linked to this article.

## Online content

Any methods, additional references, Nature Portfolio reporting summaries, source data, extended data, supplementary information, acknowledgements, peer review information; details of author contributions and competing interests; and statements of data and code availability are available at 10.1038/s41586-024-07873-4.

## Supplementary information

Supplementary Information Supplementary Figs. 1 and 2 and full descriptions for Supplementary Tables 1–15.

Reporting Summary

Supplementary Tables Supplementary Tables 1–15.

## Source data

Source Data Fig. 1

Source Data Fig. 2

Source Data Fig. 3

Source Data Fig. 4

Source Data Fig. 5

Source Data Extended Data Fig. 1

Source Data Extended Data Fig. 3

Source Data Extended Data Fig. 4

Source Data Extended Data Fig. 6

Source Data Extended Data Fig. 7

Source Data Extended Data Fig. 8

Source Data Extended Data Fig. 9

Source Data Extended Data Fig. 10

Source Data Extended Data Fig. 11

Source Data Extended Data Fig. 12

## Data Availability

The bulk RNA-seq datasets have been deposited at the Gene Expression Omnibus under the SuperSeries accession number GSE268813. The raw data from EM have been deposited in the Cell Image Library (http://cellimagelibrary.org/groups/57187). The MS proteomics data have been deposited at the ProteomeXchange Consortium via the PRIDE partner repository with the dataset identifier PXD049692. Human type I interferon network is at WikiPathways (https://www.wikipathways.org/instance/WP4868). Macrophage scRNA-seq data used from ref. ^21^ were obtained from GSE229376. The structures are available for fibrinogen (3GHG) and for spike (6VSB) were obtained from the PDB. All other data are available in the paper. Source data are provided with this paper.
