# Supplementary material for: Fibrin drives thromboinflammation and neuropathology in COVID-19
Source: Nature. 2024 Aug 28;633(8031):905–13. doi: 10.1038/s41586-024-07873-4 (PMC11424477; doi:10.1038/s41586-024-07873-4)

nature portfolio

https://doi.org/10.1038/s41586-024-07873-4

Supplementary information

Fibrin drives thromboinflammation and neuropathology in COVID-19

In the format provided by the authors and unedited

Supplementary Information

Fibrin drives thromboinflammation and neuropathology in COVID-19

Jae Kyu Ryu, Zhaoqi Yan, Mauricio Montano, Elif G. Sozmen, Karuna Dixit, Rahul K. Suryawanshi, Yusuke Matsui, Ekram Helmy, Prashant Kaushal, Sara K. Makanani, Thomas J. Deerinck, Anke Meyer-Franke, Pamela E. Rios Coronado, Troy N. Trevino, Min-Gyoung Shin, Reshmi Tognatta, Yixin Liu, Renaud Schuck, Lucas Le, Hisao Miyajima, Andrew S. Mendiola, Nikhita Arun, Brandon Guo, Taha Y. Taha, Ayushi Agrawal, Eilidh MacDonald, Olive Aries, Aaron Yan, Olivia Weaver, Mark A. Petersen, Rosa Meza Acevedo, Maria del Pilar S. Alzamora, Reuben Thomas, Michela Traglia, Valentina L. Kouznetsova, Igor F. Tsigelny, Alexander R. Pico, Kristy Red-Horse, Mark H. Ellisman, Nevan J. Krogan, Mehdi Bouhaddou, Melanie Ott, Warner C. Greene, Katerina Akassoglou

Supplementary Figures

Supplementary Fig. 1. Uncut gels. Unedited immunoblots corresponding to the indicated figures.

Supplementary Fig. 2. FACS gating strategy.

Supplementary Tables

Supplementary Table 1. Related to Fig. 1e, Peptide array mapping of fibrinogen chains Aα, Bβ, and γ blotted with Spike.

Supplementary Table 2. Related to Extended Data Fig. 2, The intermolecular interactions of the fibrinogen-Spike complex by LigPlot analysis.

Supplementary Table 3. Related to Extended Data Fig. 2, The intermolecular interactions of the fibrinogen-Spike complex by LigPlot analysis.

Supplementary Table 4. Related to Fig. 2, Differentially expressed genes (DEG) in lungs from *Fga*$^{-/-}$ vs WT mice infected with SARS-CoV-2 Beta by unbiased transcriptomics.

Supplementary Table 5. Related to Fig. 2, Significantly altered pathways in lungs from *Fga*$^{-/-}$ vs WT mice infected with SARS-CoV-2 Beta by unbiased GSEA pathway analysis.

Supplementary Table 6. Related to Fig. 3, Significant differentially expressed genes (DEG) from bulk RNA-seq analysis of NK cells stimulated with fibrin *in vitro*.

Supplementary Table 7. Related to Fig. 3, Significant pathways of bulk RNA-seq of NK cells stimulated with fibrin in vitro determined by GSEA analysis.

Supplementary Table 8. Related to Fig. 3c and Extended Data Fig. 7c, Kinase activities sheet and Phosphorylation site statistics sheet.

Supplementary Table 9. Related to Fig. 3c, Kinase activities in fibrin-stimulated NK cells.

Supplementary Table 10. Related to Extended Data Fig. 7b, Phosphorylation sites in fibrin-stimulated NK cells.

Supplementary Table 11. Related to Fig. 3e and Extended Data Fig. 8, NK1.1 antibody treatment in SARS-CoV-2 Beta-infected WT, *Fga*$^{-/-}$, or *Fgg*$^{\gamma 390\text{-}396A}$ mice.

Supplementary Table 12. Related to COVID models utilized in this study.

Supplementary Table 13. Related to Fig 5 and Extended Data Fig. 10, 11,12 Fibrin antibody treatment in WT mice infected with SARS-CoV-2 Beta, K18-hACE2 mice infected with SARS-CoV-2 B.1.617.2 Delta, or WT mice injected with Spike PVs.

Supplementary Table 14. Related to Fig. 5m, Significant differentially expressed genes (DEG) in brains from K18-hACE2 mice infected with Delta and treated with fibrin antibody.

Supplementary Table 15. Related to Extended Data Fig. 12e, Significant gene pathways in brains from K18-hACE2 mice infected with Delta and treated with fibrin antibody by clusterProfiler.

Supplementary Figure 1. Uncut gels

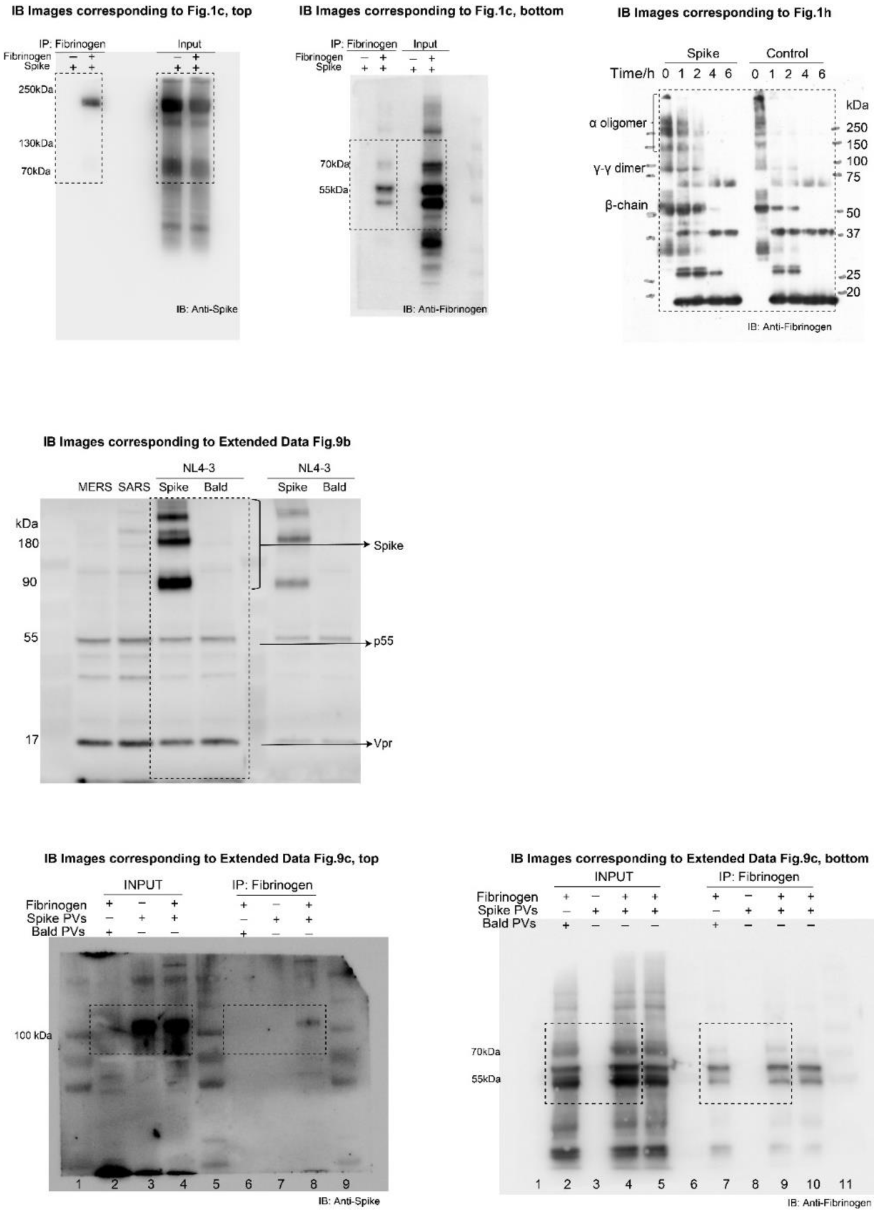

IB images corresponding to Extended Data Fig. 9b: The immune blot was performed with the sequential incubation of the following antibodies: anti-Spike, anti-p24 Gag (detecting p55), and anti-Vpr.

IB images corresponding to Extended Data Fig. 9c (top): Lanes 1, 5, 9: protein markers.

IB images corresponding to Extended Data Fig. 9c (bottom): Lanes 1, 6, 11: protein markers. Spike PVs co-immunoprecipitated with fibrinogen were confirmed using two virus concentrations in lanes 9 and 10.

Supplementary Figure 2. Gating Strategy

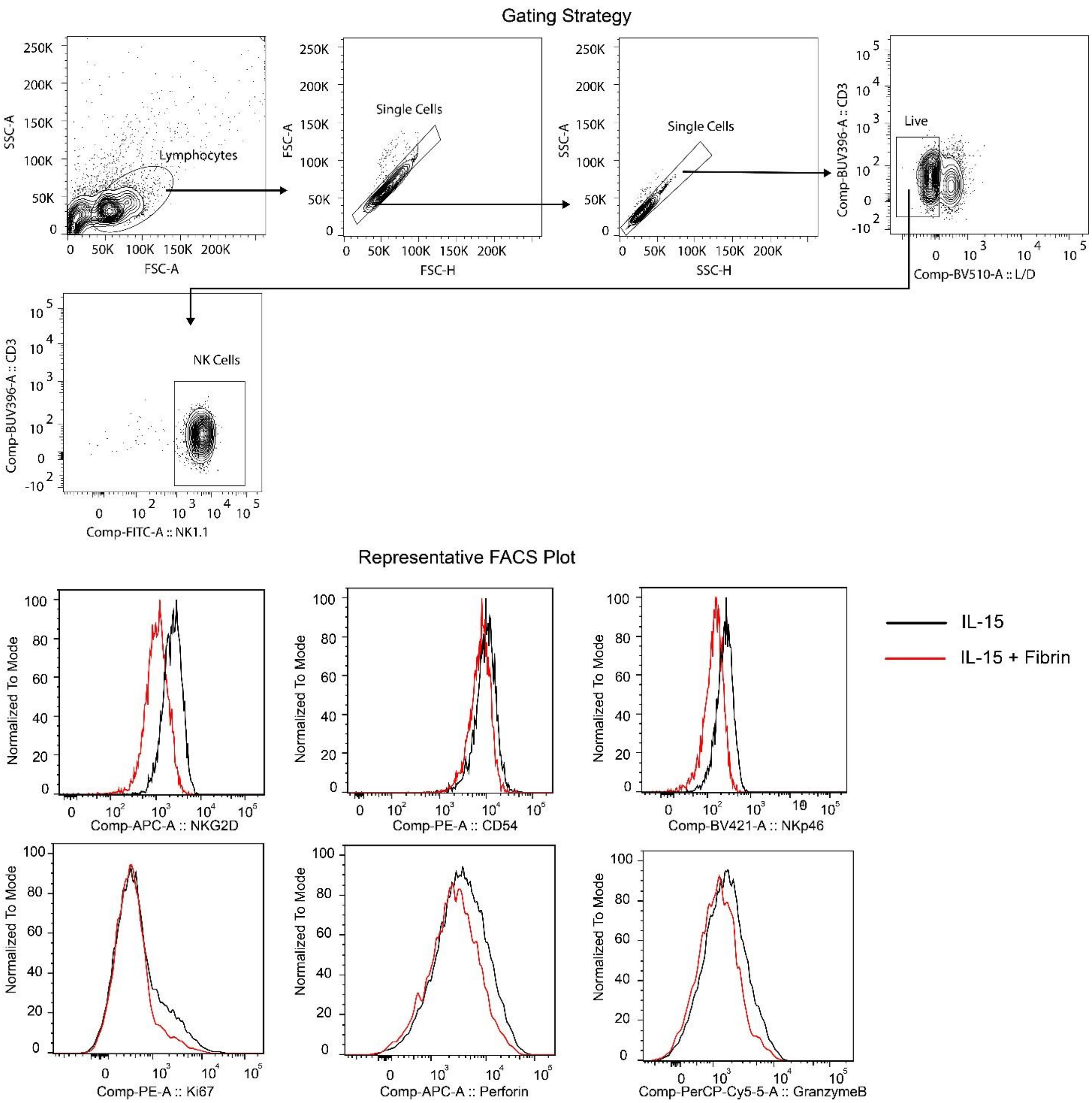

Supplement: Supplementary file 1 — Supplementary Figs. 1 and 2 and full descriptions for Supplementary Tables 1–15. [file 41586_2024_7873_MOESM1_ESM.pdf]
