# Supplementary material for: Fibrin drives thromboinflammation and neuropathology in COVID-19
Source: Nature. 2024 Aug 28;633(8031):905–13. doi: 10.1038/s41586-024-07873-4 (PMC11424477; doi:10.1038/s41586-024-07873-4)
Supplement: Supplementary file 2 — Reporting Summary [file 41586_2024_7873_MOESM2_ESM.pdf]

nature portfolio

Corresponding author(s): Katerina Akassoglou
Warner Greene

Last updated by author(s): Jun 27, 2024

Reporting Summary

Nature Portfolio wishes to improve the reproducibility of the work that we publish. This form provides structure for consistency and transparency in reporting. For further information on Nature Portfolio policies, see our Editorial Policies and the Editorial Policy Checklist.

Statistics

For all statistical analyses, confirm that the following items are present in the figure legend, table legend, main text, or Methods section.

n/a	Confirmed	
☐	☒	The exact sample size (*n*) for each experimental group/condition, given as a discrete number and unit of measurement
☐	☒	A statement on whether measurements were taken from distinct samples or whether the same sample was measured repeatedly
☐	☒	The statistical test(s) used AND whether they are one- or two-sided<br>*Only common tests should be described solely by name; describe more complex techniques in the Methods section.*
☒	☐	A description of all covariates tested
☐	☒	A description of any assumptions or corrections, such as tests of normality and adjustment for multiple comparisons
☐	☒	A full description of the statistical parameters including central tendency (e.g. means) or other basic estimates (e.g. regression coefficient) AND variation (e.g. standard deviation) or associated estimates of uncertainty (e.g. confidence intervals)
☐	☒	For null hypothesis testing, the test statistic (e.g. *F*, *t*, *r*) with confidence intervals, effect sizes, degrees of freedom and *P* value noted<br>*Give P values as exact values whenever suitable.*
☒	☐	For Bayesian analysis, information on the choice of priors and Markov chain Monte Carlo settings
☒	☐	For hierarchical and complex designs, identification of the appropriate level for tests and full reporting of outcomes
☒	☐	Estimates of effect sizes (e.g. Cohen's *d*, Pearson's *r*), indicating how they were calculated

Our web collection on statistics for biologists contains articles on many of the points above.

Software and code

Policy information about availability of computer code

Data collection

Data collection code was not used in this study

Data analysis

For SEM experiments, 4000X images were captured across the sample, then were converted to 8-bit with NIH ImageJ (v. 1.50). After pixel to micron scaling, each image was cropped into two or three fields of view (FOV) (8×8 µm) with NIH DiameterJ as described (Hotaling, et al., Biomaterials, 61, 327-338 (2015)). Surface plot plug-in ImageJ generated topographical maps of SEM images. Briefly, the best segmentation algorithm was pre-selected based on side-by-side comparison of images before quantification. The Mixed Segmentation (M1 through M3) built in DiameterJ Segment provided the most accurate representation of the fibers to be quantified. The same segmentation method and variant was used across all test conditions and images. Each segmented image was manually edited with ImageJ to ensure complete representation of segmented fibers. Edited images were batch processed with DiameterJ 1-108 (orientation analysis not selected). Fiber radius and intersection densities were collated from each batch. Data from 8–10 FOVs per sample was used for group analysis. “Fiber radius distribution” in Fig. 1f was calculated using FOVs from all images collected to assess the distribution across the dataset. “Fiber radius proportion” was statistically analyzed based on three biologically independent experiment in Fig. 1f and the quantification and statistical analysis of the individual images from these experiments is shown in Extended Data Fig. 3c. Samples with collapsed fibers due to potential SEM critical point drying technical artifact were excluded from further analysis. For quantification of the fibrin clots by SEM, at each radius, the difference in log odds of detecting fibers (among all the views in a given image) with the chosen radius under Spike versus control conditions was estimated across all images (log odds ratio). The log odds ratio at each radius is estimated using generalized linear mixed effects models, with the family argument set to binomial and implemented in glmer function in the lme4 (v1.1-27) package in R (Bates et al., J Stat Softw 67, 1 - 48, (2015)), in which the image source for the observations is modeled as a random effect. The P values were corrected for multiple testing using the Holm procedure (Holm et al., Scand J Stat 6, 65-70, (1979)).

For structure preparation and homology modeling, Crystal structure of human fibrinogen (PDB: 3GHG) was fixed using Structure Preparation

application of Compute module of MOE. is a trimeric protein. The crystal structure of SARS-CoV-2 Spike (PDB: 6VSB) has missing structural information for flexible loops. To correct these, the Homology Model application in Protein menu of MOE 2022.02 software (Chemical Computing Group ULC) was used, which includes: (1) initial partial geometry specification; (2) insertions and deletions; (3) loop selection and sidechain packing; and (4) final model selection and refinement. Homology models were inspected using MOE's Protein Geometry stereochemical quality evaluation tools. Spike crystal structure (PDB: 6VSB) was prepared by assigning protonation and ionization states.After Receptor, Ligand, and docking sites we defined, parameters of Dock application of Compute module of MOE were set to: Refinement—Rigid Body, Poses—10. The application created 10 poses, analyzed output scores, ligand docking energies, and docked poses, and detected the best one; intermediate poses also are saved in a docking database file. During the docking calculations the program presents 10 best energy complexes. After that, each of the complexes undergone the additional calculations of energy. A computational alanine scan of the fibrinogen molecules in each complex was also conducted with each of the residues in fibrinogen that were experimentally substituted to alanine were computationally substituted to alanine and modeled. The best model was selected based on lowest docking energy. The computational alanine scan generated the values of correlations between all values of energy for each amino-acid substitution and experimental values of the parameter used for estimating the influence of each amino acid. The residues involved in the interaction of this computationally predicted complex were analyzed using LigPlot+, v.2.2.

For plasmin digestion of fibrin clots, fibrin digestion band intensities of each protein species (i.e., γ-γ dimer, β-chain) were analyzed with Image J and normalized to corresponding bands at the 0 h time point. The loading control for the western blot is the timepoint 0 prior to the addition of plasmin to the fibrin clot.

For Bulk RNA-seq, generation of cDNA, sequencing, QC of raw count, mapping and counting was performed as described (Mendiola.,Nat Immunol 21, 513-524, (2020), Mendiola., Nat Immunol 24, 1173-1187, (2023)).Samples used for gene expression analysis were confirmed for viral load by qPCR in lung tissue for expression of N5 specific for beta variant. Samples with poor RNA quality or no viral load were excluded from further analysis. All samples that passed RNA quality control were included in the study. A minimum of three replicates per group was used, and genes with less than 0.1 counts per million (CPM) were filtered out from the study. Normalization were then performed using calcNormFactors, and differentially expressed genes (DEGs) were determined using edgeR (Robinson.,Genome Biol 11, R25, (2010)). False discovery rate (FDR) was calculated using the Benjamini-Hochberg method. CPM of each gene was normalized across all samples to generate z-score for heatmaps of gene expression. DEGs significantly changed in uninfected mice were not included in the analysis. For pathway analysis, gene lists were ranked using log2 fold change of differentially expressed gene between two groups. Fibrin-induced macrophage scRNA-seq data were obtained from Mendiola et al.21 (GSE229376). Gene set enrichment analysis was performed using GSEA v4.2.3 with 1000 times permutation and collapsing mouse genes to the chip platform Mouse_Gene_Symbol_Remapping_Human_Orthologs_MSigDB.v7.5.1.chip. The MSigDB gene sets: H: Hallmark and C2: CP: Canonical pathways (KEGG, REACTOME, WikiPathways) were used for pathway analysis. The fibrin NK suppression network was generated using Cytoscape v.3.7.2. Using differentially altered pathway generated by GSEA (described earlier), network was visualized using default setting of EnrichmentMap.

Additional software info: R: 4.3.2. Nextflow: bedtools (2.30.0), python (3.9.5), yaml (5.4.1), custom (1.14), bioconductor-deseq2 (1.28.0), r-base (4.0.3), bioconductor-dupradar (1.18.0), fastqc(0.11.9), rsem (1.3.1), picard (2.26.10), preseq (3.1.1),Qualimap (2.2.2-dev), salmon (1.5.2), bioconductor-summarizedexperiment (1.20.0), bioconductor-tximeta (1.8.0), samtools (1.14), star (2.6.1d), stringtie (2.1.7), subread (2.0.1), cutadapt (3.4), trimgalore (0.6.7), ucsc (377), Nextflow (21.10.6), nf-core/rnaseq (3.6). edgeR workflow: edgeR (4.0.16), Limma (3.58.1), magrittr (2.0.3), org.Mm.eg.db (3.18.0), tidyverse (2.0.0), SummarizedExperiment (1.32.0). Visualization: pheatmap (1.0.12), RColorBrewer (1.1.3), ggplot2 (3.5.0), Cytoscape: 3.10.1

Mass spectrometry proteomics data acquisition. Dried phosphopeptides were resuspended in 0.1% (v/v) FA (Sigma Aldrich) in water (HPLC grade, Fisher Scientific) and analyzed on a timsTOF HT mass spectrometer (Bruker Daltonics), paired with a Vanquish Neo ultra-high pressure liquid chromatography system (Thermo Fisher Scientific). Samples were directly injected on a PepSep C18 reverse phase column (15 cm,150 µm i.d., 100 Å pore size, 1.5 µm particle size with UHP inlet, Bruker Daltonics) connected to a captive spray emitter (ZDV, 20 µm, Bruker Daltonics). Mobile phase A consisted of 0.1% (v/v) FA in water (HPLC grade, Fisher Scientific), and mobile phase B consisted of 0.1% (v/v) FA in 100% Acetonitrile (HPLC grade, Fisher Scientific). Peptides were separated on a gradient from 3% to 25% mobile phase B over 47 minutes, followed by an increase to 45% B over 8 min, then to 95% over 1 min, and held at 95% B for 4 min for column washing at a flow rate of 200 nl/min. Eluted peptides were ionized in a CaptiveSpray source (Bruker Daltonics) at 1700 V. Raw data was acquired in data-independent acquisition coupled with parallel accumulation–serial fragmentation (dia-PASEF) mode with an optimized isolation window scheme in the m/z vs ion mobility plane for phosphopeptides. The ion accumulation time and ramp times in the dual TIMS analyzer were set to 100 ms each. For dia-PASEF, in the ion mobility (1/K0) range 0.6 to 1.50 Vs cm-2, the collision energy was linearly decreased from 59 eV at 1/K0 = 1.6 Vs cm-2 to 20 eV at 1/K0 = 0.6 Vs cm-2 to collect the MS/MS spectra in the mass range 400.2 to 1399.3 Da. The estimated mean cycle time for the dia-PASEF windows was 1.38 s. The raw files were processed with Spectronaut (v18.5, Biognosys) using its library-free DIA analysis with directDIA+ (Deep) search algorithm. Carbamidomethylation (cysteine) was set as a fixed modification for database search. Acetylation (protein N-term), oxidation (methionine), and phosphorylation (serine, threonine, tyrosine) were set as variable modifications. Reviewed human protein sequences (downloaded from UniProt, October 6, 2023) were used for spectral matching. The false discovery rates for the PSM, peptide, and protein groups were set to 0.01, and the minimum localization threshold for PTM was set to zero. For MS2 level area-based quantification, the cross-run normalization option was unchecked (normalization was performed later using MSstats 4.8.7, see below), and the probability cutoff was set to zero for the PTM localization. We detected between 4000 and 7000 phosphorylated peptides per sample with an average percentage of phosphorylated to non-phosphorylated peptides of 73%.

Computational analysis of phosphoproteomics data. Quantification of phosphorylation differences was performed using artMS as a wrapper around MSstats, via functions artMS::doSiteConversion and artMS::artmsQuantification with default settings. All peptides containing the same set of phosphorylated sites were grouped and quantified together into phosphorylation site groups. One sample outlier in intensity and peptide detection was discarded before quantitative analysis; unstimulated (Mock) 1 h (PRIDE sample ID TOF01641_2_1_1683). For both phosphopeptide and protein abundance MSstats pipelines, MSstats performs normalization by median equalization, no imputation of missing values, and median smoothing to combine intensities for multiple peptide ions or fragments into a single intensity for their protein or phosphorylation site group. Lastly, statistical tests of differences in intensity between infected and control time points were performed. When not explicitly indicated, we used defaults for MSstats for adjusted P values, even in cases of n=2. By default, MSstats uses the Student's t-test for P value calculation and the Benjamini–Hochberg method of FDR estimation to adjust P values. Kinase activities were estimated using known kinase-substrate relationships from the OmniPath database. Kinase activities were inferred as a Z-score calculated using the mean log2 fold change (log2FC) of phosphorylated substrates for each kinase in terms of standard error (Z = [M - u] / SE), comparing fold changes in phosphosite measurements of the known substrates against the overall distribution of fold changes across the sample. To compare all phosphorylation sites across experimental groups as previously described Bouhaddou et al., Cell 182:685-712 (2020), a P value was also

calculated from log2 fold changes of all detected phosphorylation sites using a two-tailed Z-test method as shown in Fig. 3c, Extended Data Fig. 6b, and Supplementary Table 8-10. Network reconstruction and enrichment analysis of phosphoproteomics data was performed as described.

For Nanostring Analysis, gene expression assays were performed on the Nanostring nCounter machine with NS_Mm_HostResponse_v1.0 codeset. The raw data was processed and normalized count, p-value, and log2 fold change were generated with nSolver. For Pathway analysis, the normalized count of each gene was normalized across all samples to generate a z-score for heat maps of gene expression. Significantly downregulated genes between the 5B8 and IgG2b treated group ($P < 0.05$) were on clusterProfiler to determine significantly downregulated pathways using the enrichGO function. The top 20 significantly downregulated pathways were used to generate the network.

Additional software info: Clusterprofiler: R: 4.3.2., clusterProfiler (4.10.1), enrichplot (1.22.0), ggnewscale (0.4.10). Visualization: pheatmap (1.0.12), RColorBrewer (1.1.3), ggplot2 (3.5.0),

For image analysis, immunostained cells were counted with Jupyter Notebook in Python 3. Briefly, an arbitrary threshold was manually set and used for all images in the dataset. The total number of cells per image was estimated with the function “peak_local_max” from the open source “skimage” Python image processing library, which returns the coordinates and number of local peaks in an image (https://scikit-image.org/docs/dev/api/skimage.feature.html#skimage.feature.peak_local_max). Fibrinogen immunoreactivity was quantified with Fiji (ImageJ) as described (Davalos., Nat Commun 3, 1227, (2012)). Python image processing was used to colocalize fibrinogen and Spike protein in lung tissues. Briefly, a Jupyter Notebook was written to estimate the amount of fluorescent signal overlap between Spike and fibrinogen in lung tissues. The “Ostu” filter from the “skimage” Python image processing library was used to threshold each image labeled with Spike and fibrinogen (https://scikit-image.org/docs/0.13.x/api/skimage.filters.html#skimage.filters.threshold_otsu). After thresholding, each set of images was compared, and pixels were compartmentalized in 4 categories: Spike and fibrinogen overlap, Spike signal only, fibrinogen signal only, and no signal. In each image, the total number of pixels in an image and the number of pixels with signal for Spike only, fibrinogen only, or both were computed. Correlations were calculated using FOVs from all images collected as indicated in Extended Figs. 1b, c and 8f to assess the distribution across the dataset. All images selected for the Figures are representative of the quantification of immunostaining for each experimental group.

For manuscripts utilizing custom algorithms or software that are central to the research but not yet described in published literature, software must be made available to editors and reviewers. We strongly encourage code deposition in a community repository (e.g. GitHub). See the Nature Portfolio guidelines for submitting code & software for further information.

Data

Policy information about availability of data

All manuscripts must include a data availability statement. This statement should provide the following information, where applicable:

- Accession codes, unique identifiers, or web links for publicly available datasets
- A description of any restrictions on data availability
- For clinical datasets or third party data, please ensure that the statement adheres to our policy

The bulk RNA-seq datasets are deposited in the Genome Expression Omnibus under the SuperSeries accession number GSE268813. The raw data from electron microscopy are deposited in the Cell Image Library at http://cellimagelibrary.org/groups/57187. The mass spectrometry proteomics data have been deposited to the ProteomeXchange Consortium via the PRIDE partner repository with the dataset identifier PXD049692. Human type I interferon network is at WikiPathways https://www.wikipathways.org/instance/WP4868. Macrophage scRNA-seq data were obtained from GSE229376 (Mendiola et al., Nat Immunol 24, 1173-1187 (2023)). The structures are available for fibrinogen at PDB ID:3GHG and for Spike at PDB ID:6VSB. All other data are available in the paper. Source Data are provided with this paper.

Research involving human participants, their data, or biological material

Policy information about studies with human participants or human data. See also policy information about sex, gender (identity/presentation), and sexual orientation and race, ethnicity and racism.

Reporting on sex and gender	N/A
Reporting on race, ethnicity, or other socially relevant groupings	N/A
Population characteristics	N/A
Recruitment	All human material used in the study is commercially available and no human subjects were recruited. Human citrated plasma (Catalogue number: IPLASEATNAC50ML; Lot No 1151254) was purchased from Innovative Research (Novi, MI). Fresh PBMCs (Catalogue number: LP,FR,MNC,2B; Lot No 3118730 and 3112992) were purchased from AllCells (Alameda, CA).
Ethics oversight	N/A

Note that full information on the approval of the study protocol must also be provided in the manuscript.

Field-specific reporting

Please select the one below that is the best fit for your research. If you are not sure, read the appropriate sections before making your selection.

☒ Life sciences ☐ Behavioural & social sciences ☐ Ecological, evolutionary & environmental sciences

For a reference copy of the document with all sections, see nature.com/documents/nr-reporting-summary-flat.pdf

Life sciences study design

All studies must disclose on these points even when the disclosure is negative.

Sample size

No statistical methods were used to pre-determine sample sizes but sample sizes are similar to our previous publications (Ryu et al., Nat Commun 6, 8164 (2015); Ryu et al., Nat Immunol., 19, 1212-1223 (2018); Suryawanshi., et al., Nature, 607, 351-355 (2022); Mendiola et al. Nat Immunol., 24, 1173–1187 (2023).

Data exclusions

For Bulk RNA-seq analysis and phosphoproteomics data analysis, samples were excluded from further analysis if they did not pass quality control. For Lung Bulk RNA-seq analysis, two samples were removed that did not pass RNA and cDNA library quality control testing. One sample was removed due to no viral RNA detection. For phosphoproteomics data analysis, one sample outlier in intensity and peptide detection was discarded before quantitative analysis; Mock 1 h (PRIDE sample ID TOF01641_2_1_1683). For fibrin clot SEM analysis, samples with collapsed fibers due to potential SEM critical point drying technical artifact were excluded from further analysis. No other samples or animals were excluded from any other analyses.

Replication

The number of experimental repeats is detailed at the bottom of each legend for each figure. All attempts at replication following the protocol described in the methods were successful. Biochemical studies of the binding of fibrinogen/fibrin to Spike were performed in the Akassoglou lab and independently validated in the Greene lab and Assay Development and Drug Discovery Core at the Gladstone Institutes with similar results.

Randomization

For all in vivo infection studies, mice were randomized by sex, age and genotype and blindly coded for group assignment and data collection. For antibody treatments, mice were randomized by sex and age and blindly coded for group assignment to blinded antibody administration and data collection. For in vivo stereotactic injections, age-matched and sex-matched mice were blindly coded for group assignment and data collection. For PV experiments, sex-matched mice were randomized by age and blindly coded for group assignment to blinded antibody administration and data collection. For experiments other than mice, every treatment condition included all samples, randomization was therefore not relevant.

Blinding

All in vivo experimenst were conducted in a blinded manner to the mouse genotype, antibody or PV administration. All images were acquired and quantified by an observer blinded to experimental conditions. Genotype and treatment assignment were revealed after completion of all image quantification. Bulk RNA-seq experiments were performed in a blinded manner for both mouse genotype and antibody administration. Plaque assays were carried out blinded to experimental groups. SEM image acquisition and quantification were carried out blinded to test conditions. Blinding was not applied to RNAScope quantification, because the two groups were easily identified due to the obvious difference in Iba-1 signal between infected and uninfected mice. Blinding was not applied to the following experiments with unbiased automated data acquisition and analysis using the same equipment and software settings reducing the likelihood of bias in final readouts: biochemical studies, ROS assay, flow cytometry data acquisition and analysis, mass spectrometry proteomics data acquisition and analysis, NanoString analysis.

Reporting for specific materials, systems and methods

We require information from authors about some types of materials, experimental systems and methods used in many studies. Here, indicate whether each material, system or method listed is relevant to your study. If you are not sure if a list item applies to your research, read the appropriate section before selecting a response.

Materials & experimental systems

n/a	Involved in the study
☐	☒ Antibodies
☐	☒ Eukaryotic cell lines
☒	☐ Palaeontology and archaeology
☐	☒ Animals and other organisms
☒	☐ Clinical data
☒	☐ Dual use research of concern
☒	☐ Plants

Methods

n/a	Involved in the study
☒	☐ ChIP-seq
☐	☒ Flow cytometry
☒	☐ MRI-based neuroimaging

Antibodies

Antibodies used

Immunohistochemistry: rabbit anti-SARS-CoV-2 nucleocapsid (GTX135357, GeneTex; 1:500), mouse anti-SARS-CoV-2 Spike (1A9, GeneTex; 1:100), sheep anti-fibrinogen (F4200-06, US Biological; 1:300), rabbit polyclonal anti-fibrinogen (gift from Dr. Jay Degen not commercially available generated at his laboratory at the University of Cincinnati, OH; 1:500), rat anti-mouse/human Mac-2 (M3/38, Cedarlane; 1:500), mouse anti-gp91-phox (53/gp91-phox, BD Biosciences; 1:500), rat anti-mouse CD335 (29A1.4, BD

Biosciences; 1:500), rabbit anti-Granzyme A (PA5-119160, Invitrogen; 1:500), rabbit anti-Iba-1 (#019–19741, Wako; 1:1,000), rat anti-myelin basic protein (ab7349, Abcam; 1:100), and rabbit anti-Calbindin (Swant; CB38a, 1:5000). Secondary: goat anti-rabbit Alexa Fluor 488 (1:1000, Thermo Fisher Scientific, A-11008), goat anti-mouse Alexa Fluor 568 (A-110041, Thermo Fisher Scientific; 1:1000), or goat anti-rat Alexa Fluor 647 (A-21247, Thermo Fisher Scientific; 1:1000).

Flow cytometry: NK1.1-FITC (clone S17016D, Biolegend, 1:200), IFN-g (clone XMG1.2, Biolegend, 1:200), Granzyme B-PerCP/Cy5.5 (clone QA16A02, Biolegend, 1:200), Ki-67-PE (clone 16A8, Biolegend,1:200), CD45-Brilliant Violet BUV737(30-F11, BD, 1:200), CD11b-Brilliant Ultraviolet 395 (M1/70, BD, 1:200), CD335-Brilliant Violet 421 (Clone 29A1.4, Biolegend,1:100), CD54-PE (Clone YN1/1.7.4, Biolegend, 1:200), CD314-APC (Clone CX5, Biolegend, 1:200), IFN-γ (Clone XMG1.2, Biolegend, 1:200), anti-CD3 (Clone 145-2C11, BD, 1:200)

NK Depletion Experiment: anti-NK1.1 (Clone PK136, BioXCell), isotype control IgG2a (clone C1.18.4, BioXcell), 8mg/kg

Spike protein binding on fibrin: goat anti-rabbit IgG H&L (conjugated with horse radish peroxidase, HRP) (ab205718, Abcam, 1:1000),

Peptide Array: anti-6xHis monoclonal antibody (Alexa 647, Invitrogen, MA1-135-A647, 1 µg/ml final concentration)

Plasma digestion of fibrinogen: sheep anti-fibrinogen (F4200-06 (now available as catalogue number F4203-02F), US Biological; 1:300))

Competitive ELISA: anti-His tag antibody (R&D Systems, MAB050H, 1:2,000)

Immunoprecipitation and Immunoblot: anti-fibrinogen antibody (1:1000, Enzyme Research Laboratories, SAFG-AP), anti-Spike antibody (1:1000, GeneTex, 632604) , sheep anti-fibrinogen antibody (1:1000, Enzyme Research Laboratories, SAFG-AP), anti-Spike (632604, GeneTex, 1:1000), anti-p24 Gag (detecting p55, 1:100) donated to the Greene lab by Beckman Coulter not commercially available and anti-Vpr (8D1, Cosmo Bio, 1:200), HRP-conjugated anti-rabbit (111-035-144, Jackson ImmunoResearch; 1:10,000), Sheep IgG HRP-conjugated Ab (HAF016, R&D Systems; 1:5000) secondary antibodies

Validation

Immunohistochemistry:
rabbit anti-SARS-CoV-2 nucleocapsid (GTX135357, GeneTex; 1:500), https://www.genetex.com/Product/Detail/SARS-CoV-2-COVID-19-Nucleocapsid-antibody/GTX135357

mouse anti-SARS-CoV-2 Spike (Clone 1A9, GTX632604, GeneTex; 1:100), https://www.genetex.com/Product/Detail/SARS-CoV-SARS-CoV-2-COVID-19-spike-antibody-1A9/GTX632604

sheep anti-fibrinogen (F4200-06 (now available as catalogue number F4203-02F), US Biological; 1:300), https://www.usbio.net/antibodies/F4203-02F/Fibrinogen-Coagulation-Factor-I

rabbit polyclonal anti-fibrinogen (gift from Dr. Jay Degen, not commercially available generated at his laboratory at the University of Cincinnati, OH; University of Cincinnati, OH; 1:500). Antibody specificity for fibrinogen was validated in fibrinogen KO mouse tissue in Wang J et al., World J Gastroenterol. Jul 14;23(26):4701-4711 (2017).

rat anti-mouse/human Mac-2 (Clone M3/38, CL8942AP, Cedarlane; 1:500), https://www.cedarlanelabs.com/Products/Detail/CL8942AP?lob=AllProducts

mouse anti-gp91-phox (Clone 53/gp91-phox, 611414, BD Biosciences; 1:500), https://www.bdbiosciences.com/en-ca/products/reagents/microscopy-imaging-reagents/immunofluorescence-reagents/purified-mouse-anti-gp91-phox.611414

rat anti-mouse CD335 (Clone 29A1.4, 560754, BD Biosciences; 1:500), https://www.bdbiosciences.com/en-us/products/reagents/flow-cytometry-reagents/research-reagents/single-color-antibodies-ruo/purified-rat-anti-mouse-cd335-nkp46.560754

rabbit anti-Granzyme A (PA5-119160, Invitrogen; 1:500), https://www.thermofisher.com/antibody/product/Granzyme-A-Antibody-Polyclonal/PA5-119160

rabbit anti-Iba-1 (019–19741, Wako; 1:1,000), https://labchem-wako.fujifilm.com/us/product/detail/W01W0101-1974.html

rat anti-myelin basic protein (ab7349, Abcam; 1:100),
https://www.abcam.com/products/primary-antibodies/myelin-basic-protein-antibody-12-ab7349.html

rabbit anti-Calbindin (CB38a, Swant; 1:5000), https://webshop.swant.com/cb38a-calbindin.html

Flow cytometry:

NK1.1-FITC (Clone S17016D, 156507, Biolegend), https://www.biolegend.com/en-gb/products/fitc-anti-mouse-nk-11-antibody-19869

IFN-g-PE (Clone XMG1.2, Biolegend), https://www.biolegend.com/fr-ch/products/pe-anti-mouse-ifn-gamma-antibody-997

Granzyme B-PerCP/Cy5.5 (Clone QA16A02, 372211, Biolegend), https://www.biolegend.com/nl-nl/products/percp-cyanine5-5-anti-humanmouse-granzyme-b-recombinant-antibody-15597

Ki-67-PE (Clone 16A8, 652403, Biolegend), https://www.biolegend.com/de-at/products/pe-anti-mouse-ki-67-antibody-8134

CD45-Brilliant Violet BUV737(Clone 30-F11, 748371, BD Biosciences), https://www.bdbiosciences.com/en-us/products/reagents/flow-cytometry-reagents/research-reagents/single-color-antibodies-ruo/buv737-rat-anti-mouse-cd45.748371

CD11b-Brilliant Ultraviolet 395 (Clone M1/70, 565976, BD Biosciences), https://www.bdbiosciences.com/en-us/products/reagents/flow-cytometry-reagents/research-reagents/single-color-antibodies-ruo/buv395-rat-anti-cd11b.565976

CD335-Brilliant Violet 421 (Clone 29A1.4, 137611, Biolegend), https://www.biolegend.com/en-us/products/brilliant-violet-421-anti-mouse-cd335-nkp46-antibody-7506

CD54-PE (Clone YN1/1.7.4, 116107, Biolegend), https://www.biolegend.com/en-gb/products/pe-anti-mouse-cd54-antibody-1680

CD314-APC (Clone CX5, 130211, Biolegend), https://www.biolegend.com/en-gb/clone-search/apc-anti-mouse-cd314-nkg2d-antibody-5267?GroupID=BLG10492

NK Depletion Experiment:
anti-NK1.1 (Clone PK136, BP0036, BioXCell), https://bioxcell.com/invivoplus-anti-mouse-nk1-1-bp0036
isotype control IgG2a (Clone C1.18.4, BP0085, BioXcell), https://bioxcell.com/invivoplus-mouse-igg2a-isotype-control-unknown-specificity

Validation of the anti-mouse NK1.1 (PK136, BioXCell), which depletes NK cells was also reported in the following publications (Pollenus et al., Malar J 23, 110, (2024), Burrack et al., Immunity 48, 760-772 e764, (2018), Wensveen et al., Nat Immunol 16, 376-385, (2015).)

Spike protein binding on fibrin:
goat anti-rabbit IgG H&L (conjugated with horse radish peroxidase, HRP) (ab205718, Abcam, 1:1000), https://www.abcam.com/products/secondary-antibodies/goat-rabbit-igg-hl-hrp-ab205718.html

Peptide Array:
Peptide Array: anti-6xHis monoclonal antibody (Alexa 647, Invitrogen, MA1-135-A647, 1 µg/ml final concentration), https://www.thermofisher.com/antibody/product/6x-His-Tag-Antibody-clone-4E3D10H2-E3-Monoclonal/MA1-135-A647

Plasma digestion of fibrinogen: anti-human fibrinogen (US Biological, F4200-06, 1:2000)
Discontinued.

Competitive ELISA: anti-His tag antibody (R&D Systems, MAB050H, 1:2,000), https://www.rndsystems.com/products/his-tag-horseradish-peroxidase-conjugated-antibody-ad1110_mab050h

Immunoprecipitation and Immunoblot
Sheep anti-fibrinogen antibody (1:1000, Enzyme Research Laboratories, SAFG-AP), https://enzymeresearch.com/wp-content/uploads/2019/09/SAFG-AP-sample-CofA.pdf
anti-Spike antibody (1:1000, GeneTex, 632604) , https://www.genetex.com/Product/Detail/SARS-CoV-SARS-CoV-2-COVID-19-spike-antibody-1A9/GTX632604
anti-Spike antibody (1:1600, GeneTex, 635693) https://www.genetex.com/Product/Detail/SARS-CoV-2-COVID-19-Spike-S2-antibody-HL237/GTX635693
anti-p24 Gag (detecting p55, 1:100) donated to the Greene lab by Beckman Coulter not commercially available validated in Stopak et al., Mol Cell 12: (3) 591-601 (2003).
anti-Vpr (8D1, Cosmo Bio, 1:200) https://www.cosmobiousa.com/products/anti-vpr-hiv-1-mab-clone-8d1

Eukaryotic cell lines

Policy information about cell lines and Sex and Gender in Research

Cell line source(s)	293T cells were obtained from ATCC and used for protein production. Vero cells expressing TMPRSS and ACE2 (Vero-TMPRSS2-ACE2) were provided by A. Creanga and B. Graham at NIH and cultured in DMEM supplemented with 10% FBS and blasticidin (20µg ml−1) (Sigma) at 37°C and 5% CO_2. CHO cells were cultured and used for protein production at Celltheon (Union City, CA).
Authentication	Protein expression was confirmed with western blot. No further authentication was conducted.
Mycoplasma contamination	Annual mycoplasma contamination tests are performed on all cell lines and all the cell line were found negative for mycoplasma contamination.
Commonly misidentified lines (See ICLAC register)	No commonly misidentified cell lines were used in current study.

Animals and other research organisms

Policy information about studies involving animals; ARRIVE guidelines recommended for reporting animal research, and Sex and Gender in Research

Laboratory animals	C57BL/6 mice and K18-hACE2 mice (strain: B6.Cg-Tg(K18-ACE2)2Prlmn/J) were purchased from the Jackson Laboratory. Fga-/- and Fggγ390–396A mice were obtained from Dr. Jay Degen (University of Cincinnati, OH, USA).

Wild animals	The study did not involve wild animals
Reporting on sex	Both male and female mice were used in experiments
Field-collected samples	The study did not involve samples collected from the field
Ethics oversight	Mice were housed under a 12:12 light/dark cycle, 55% ± 5% relative humidity, and a temperature of 20 ± 2 °C with access to standard laboratory chow and water ad libitum. They were housed in social groups of a maximum of 5 mice in standard mouse housing cages and bedding. All single -housed mice were provided with cage enrichment (a cardboard or hard-plastic house-like hiding place and tissue paper). For husbandry, one male and one female were housed together with a maximum of one litter was permitted. Mice were weaned at postnatal day 21. Infection experiments were performed at a AAALAC accredited ABSL3 facility at Gladstone Institutes. All animal procedures were performed under the guidelines set by the Institutional Animal Care and Use Committee at the University of California, San Francisco.

Note that full information on the approval of the study protocol must also be provided in the manuscript.

Plants

Seed stocks	*Report on the source of all seed stocks or other plant material used. If applicable, state the seed stock centre and catalogue number. If plant specimens were collected from the field, describe the collection location, date and sampling procedures.*
Novel plant genotypes	*Describe the methods by which all novel plant genotypes were produced. This includes those generated by transgenic approaches, gene editing, chemical/radiation-based mutagenesis and hybridization. For transgenic lines, describe the transformation method, the number of independent lines analyzed and the generation upon which experiments were performed. For gene-edited lines, describe the editor used, the endogenous sequence targeted for editing, the targeting guide RNA sequence (if applicable) and how the editor was applied.*
Authentication	*Describe any authentication procedures for each seed stock used or novel genotype generated. Describe any experiments used to assess the effect of a mutation and, where applicable, how potential secondary effects (e.g. second site T-DNA insertions, mosiacism, off-target gene editing) were examined.*

Flow Cytometry

Plots

Confirm that:

☒ The axis labels state the marker and fluorochrome used (e.g. CD4-FITC).

☒ The axis scales are clearly visible. Include numbers along axes only for bottom left plot of group (a 'group' is an analysis of identical markers).

☒ All plots are contour plots with outliers or pseudocolor plots.

☒ A numerical value for number of cells or percentage (with statistics) is provided.

Methodology

Sample preparation	Sample preparation for mass spectrometry. Human NK cells were isolate from freshly collect PBMC from three individual (AllCells) using NK cell Isolation Kit, Human (Miltenyi Biotec, 130-092-657). 5 x 10^6 NK cells were plated on each well of 6 well plate treated with or without fibrin for one hour at 37 ºC. Phosphoproteomic analysis was performed as described22,35. Samples were washed 2x with cold PBS, lysed in 6M guanidine hydrochloride (Sigma Aldrich), then boiled at 95°C for 5 minutes, and stored on ice until sonication. Lysed samples were sonicated using a probe sonicator 1x for 15 seconds at 10% amplitude, and protein was quantified using a Bradford assay. Approximately 500µg of protein sample was used for further processing, starting with reduction and alkylation using a 1:10 sample volume of tris-(2-carboxyethyl) (TCEP) (10 mM final) and 2-chloroacetamide (40 mM final) for 5 minutes at 45°C with shaking at 1500rpm. Before protein digestion, the 6M guanidine hydrochloride was diluted 6-fold with 100mM Tris-HCL (pH 8) to permit the activity of the proteolytic enzyme trypsin. Trypsin was then added at a 1:100 (wt/wt) enzyme-substrate ratio and placed in a thermomixer at 37°C overnight (16 hours) with shaking at 800rpm. Following digestion, 10% trifluoroacetic acid (TFA) was added to each sample to reach a final pH of 2. Samples were then desalted using a vacuum manifold with 50mg Sep Pak C18 cartridges (Waters). Each cartridge was activated with 1 mL 80% acetonitrile (ACN)/0.1% TFA, then equilibrated with 3 x 1 mL of 0.1% TFA. After sample loading, cartridges were washed with 3 x 1 mL of 0.1% TFA, and samples were eluted with 1 x 0.8 mL 50%ACN/0.25% formic acid (FA). Samples were then dried by vacuum centrifugation. The High-Select Fe-NTA phosphopeptide enrichment kit (Thermo Fisher Scientific) was used according to the manufacturer's instructions with minor modifications for phosphopeptide enrichment. Briefly, samples were suspended in approximately one-third of the recommended binding/wash buffer volume (70µL). After equilibrating the spin column, the resin slurry was resuspended in 210µL of binding/wash buffer and divided into thirds. Each third of the resin was used for one sample. Tryptic peptides were mixed with the resin in a separate protein LoBind tube (Eppendorf) and incubated for 30 min (RT) on a thermomixer at 800 rpm. Samples were then transferred on top of a 20µL filtered tip, washed three times with the provided binding/wash buffer, and once with HPLC grade water. The bound phosphopeptides were eluted with 70µL elution buffer, and the pH was brought down immediately to nearly three with formic acid (10% (v/v) in HPLC grade water). All samples were dried by vacuum centrifugation and stored at -80°C until further analysis.<br><br>Sample preparation for Flow cytometry. NK cells were purified from the splenocytes of C57BL/6 mice using the NK cell isolation kit (Miltenyi Biotec). To assess fibrin-mediated NK-cell effector function, NK cells were stimulated with IL-15 (50 ng/

ml, Biolegend) for 4 days with or without fibrin. Flow cytometric staining and analyses were performed following established guidelines. For NK cell surface and intracellular staining, NK cell suspensions were first incubated with TruStain FcX™ PLUS (clone S17011E, Biolegend) for 15 min at 4 degree, then stained with surface markers for 30 min at 4 degree. Cells were then fixed and permeabilized with BD Fixation/Permeablization Kit (554714, BD). Intracellular markers were incubated for 1h at 4 degree and analyzed with LSR Fortessa flow cytometer (BD Biosciences) the same day. For IFN-γ staining, NK cells were incubated with phorbol 12-myristate 13-acetate (P8139, Sigma) and ionomycin (I0634, Sigma) for 4 h in the presence of Brefeldin A (B7651, Sigma) followed by surface staining and fixation/permeabilization protocol. Anti-IFN-γ antibody were incubated in Perm/Wash buffer overnight, and then analyzed with LSR Fortessa flow cytometer (BD Biosciences) the same day. All data were processed with FlowJo v10.7.1 (BD Biosciences). Doublets and, dead cells were excluded before analysis of NK cell phenotypes. NK cells were gated as CD45+CD3-NK1.1+.

Sample preparation for bulk RNA-seq of murine NK cells. Purified NK cells from splenocytes of C57BL/6 mice were stimulated with IL-15 (50 ng/ml, Biolegend) for 4 days with or without fibrin. NK cells were staining with anti-CD3 (Biolegend), anti-NK1.1 (Clone S17016D, Biolegend), anti-CD45 (Clone 30-F11,Biolegend), and aqua live/dead fixable dye on ice for 20 min. The CD45+CD3-NK1.1+ live NK cells were sorted into 1.5 ml tube with 1 ml of Buffer RLT Plus with 1% beta-mercaptoethanol. RNA samples were prepared with RNeasy Plus Micro Kit according to the manufacturer's instructions. The cDNA library generation, QC, sequencing, and downstream analysis are performed as previously described.

Instrument

FORTESSA X-20 for analysis and BD FACS Aria Fusion for cell sorting

Software

BD FACSDiva v8 and FlowJo software v10

Cell population abundance

When cells were sorted or enriched, the purity was confirmed by flow cytometry and routinely >98 %.

Gating strategy

Single cells were gated by FSC-A/SSC-A and then doublet discrimination was performed first by FSC-H/FSC-W followed by SSC-H/SSC-W. Next, live cells were gated using aqua live/dead staining, NK cells are gated as CD45+CD3-NK1.1+.

☒ Tick this box to confirm that a figure exemplifying the gating strategy is provided in the Supplementary Information.
